# Performance and milk quality parameters of Jersey crossbreds in low-input dairy systems

**DOI:** 10.1038/s41598-022-10834-4

**Published:** 2022-05-09

**Authors:** Sabrina Ormston, Hannah Davis, Gillian Butler, Eleni Chatzidimitriou, Alan W. Gordon, Katerina Theodoridou, Sharon Huws, Tianhai Yan, Carlo Leifert, Sokratis Stergiadis

**Affiliations:** 1 Department of Animal Sciences, School of Agriculture, Policy and Development, University of Reading, New Agriculture Building, Earley Gate, Reading, RG6 6EU UK; 2 School of Natural and Environmental Sciences, Newcastle University, Newcastle upon Tyne, NE1 7RU UK; 3 Residues And Food Safety Unit, Regulated Products Assessment Department, French Agency for Food Environmental and Occupational Health and Safety (ANSES), 14 rue Pierre et Marie Curie, Maisons-Alfort, France; 4 Statistical Services Branch, Agri-Food and Biosciences Institute, Newforge Lane, Belfast, BT9 5PX Co. Antrim UK; 5 Institute for Global Food Security, Queen’s University Belfast, Belfast, BT9 5DL UK; 6 Sustainable Agri-Food Sciences Division, Agri-Food and Biosciences Institute, Hillsborough, BT26 6DR Co. Down UK; 7 Department of Nutrition, IMB, University of Oslo, Sognsvannsveien, 0372 Oslo, Norway; 8 Southern Cross Plant Science, Southern Cross University, Military Road, Lismore, NSW 2480 Australia

**Keywords:** Animal breeding, Animal physiology, Nutrition

## Abstract

Previous work has demonstrated some benefit from alternative breeds in low-input dairying, although there has been no systematic analysis of the simultaneous effect of Jersey crossbreeding on productivity, health, fertility parameters or milk nutritional quality. This work aimed to understand the effects of, and interactions/interrelations between, dairy cow genotypes (Holstein-Friesian (HF), Holstein-Friesian × Jersey crossbreds (HF × J)) and season (spring, summer, autumn) on milk yield; basic composition; feed efficiency, health, and fertility parameters; and milk fatty acid (FA) profiles. Milk samples (n = 219) and breed/diet data were collected from 74 cows in four UK low-input dairy farms between March and October 2012. HF × J cows produced milk with more fat (+ 3.2 g/kg milk), protein (+ 2.9 g/kg milk) and casein (+ 2.7 g/kg milk); and showed higher feed, fat, and protein efficiency (expressed as milk, fat and protein outputs per kg DMI) than HF cows. Milk from HF × J cows contained more C4:0 (+ 2.6 g/kg FA), C6:0 (+ 1.9 g/kg FA), C8:0 (+ 1.3 g/kg FA), C10:0 (+ 3.0 g/kg FA), C12:0 (+ 3.7 g/kg FA), C14:0 (+ 4.6 g/kg FA) and saturated FA (SFA; + 27.3 g/kg milk) and less monounsaturated FA (MUFA; -23.7 g/kg milk) and polyunsaturated FA (− 22.3 g/kg milk). There was no significant difference for most health and fertility parameters, but HF × J cows had shorter calving interval (by 39 days). The superior feed, fat and protein efficiency of HF × J cows, as well as shorter calving interval can be considered beneficial for the financial sustainability of low-input dairy farms; and using such alternative breeds in crossbreeding schemes may be recommended. Although statistically significant, it is difficult to determine if differences observed between HF and HF × J cows in fat composition are likely to impact human health, considering average population dairy fat intakes and the relatively small difference. Thus, the HF × J cow could be used in low-input dairying to improve efficiency and productivity without impacting milk nutritional properties.

## Introduction

Low-input farming has become increasingly prevalent due to the associated lower production costs, improved nutritional quality and perceived sustainability and welfare^1,2^. Low-input ruminant systems are characterised by high contribution (> 80% dry matter intake (DMI) of pasture (during the grazing season) and/or conserved forage (mainly during the indoor periods), and low contribution of concentrate feeds. There is evidence that production system and feeding intensity influences milk production parameters^3,4^ and given the high contribution of fresh forage in the diets of dairy cows in low-input systems, efficient conversion of feed, in particular conserved forages and pasture, to milk, is essential^5^.

Dairy breeding programs in conventional and intensive production systems have traditionally focussed on production characteristics^5,6^. The Holstein-Friesian (HF) breed has been extensively used due to high yield capabilities and is the most common dairy breed in the UK, accounting for 78% of the total milking herd in Britain^7^. However, concerns have arisen over declining fertility, health and longevity of purebred HF cows^8^; as well as reduced efficiency under low-input management^9^. This could be due to the fact that HF cows require high quantities of concentrate feed intake to achieve their yield potential, a practice not used in low-input systems^10^. Alternatively, other breeds are often selected on the assumption they improve efficiency, robustness and fertility under low-input farming practices^4–6,11^ and, in this case, breeding choices also focus on functional traits beyond milk yield, including health and fertility characteristics^6^.

Research has identified the Jersey breed as suitable to cross with purebred HF cows, because of improved milk qualities, such as higher protein and fat content^12^, and improved reproductive performance^13^, longevity^14^, and their overall adaptation to grazing systems if managed appropriately^5,15^. Research suggests that Holstein-Friesian × Jersey crossbred (HF × J) cows have better fertility^13^, higher survival rates^16^, longevity^14^ and lower health incidences than HF cows^5,8,17^. These findings, together with milk payments schemes to compensate for higher milk fat and protein contents^18^, have subsequently resulted in the HF × J becoming common additions to dairy herds, particularly low-input systems^17^, a steady decline in purebred HF cow numbers and a shift towards crossbred cows; all factors that eventually impact milk yield and composition^7^.

Purebred HF cows have demonstrated higher milk yields^12,19^ and lower milk fat and protein contents than HF × J cows^4,12,13^. However, these differences are not consistent across all studies, with some reporting similar milk fat contents between HF and HF × J^19,20^ as well as similar milk fat and protein per unit of dry matter intake (DMI) or feed efficiency (FE)^19^. One investigation into efficiency parameters demonstrated higher milk energy output per kg live weight^0.75^ for HF × J, when compared with HF cows^17^. Unfortunately, measures of efficiency are inconsistent, with studies expressing efficiency as milk energy per kg live weight^0.75^^17^, milk solids per kg of body weight (BW)^0.75^^12^, economic efficiency^21^, feed conversion efficiency (milk yield per kg DMI), and energy corrected milk yield (kg) per kg DMI^22^, which may contribute to discrepancies when the term efficiency is used generically. Current literature lacks comparisons of FE between HF and HF × J in low-input systems. Interestingly, although FE is equally important in low-input systems as in conventional high input systems, there are also additional priorities and different strategies^5^. For example, rather than focussing on increasing output, low-input systems focus on reducing external inputs and in particular feed^2,5^.

Cow breed may also affect milk fatty acid (FA) profile^3,23^, thus affecting the nutritional properties of milk. Milk fat has approximately 70% saturated FA (SFA)^24^ which are currently overconsumed in the western diets^25^. It is recommended that overall SFA intake is reduced (to less than 10% total energy intake) due to their association with increased risk of cardiovascular diseases (CVD)^25^. However, milk also contains (i) monounsaturated FA (MUFA), such as c9 C18:1 (oleic acid, OA) and t11 C18:1 (vaccenic acid, VA) which are associated with reduced risk of CVD and (ii) polyunsaturated FA (PUFA), such as n-3, c9c12c15 C18:3 (α-linolenic acid, ALNA,), conjugated linoleic acid (CLA) and its isomer c9t11 C18:2 (rumenic acid, RA) which also reduce CVD risk, as well as CLA being associated with anticancer properties^26^, and eicosapentaenoic acid (EPA) and docosapentaenoic acid (DPA), which are associated with beneficial effects on some diseases and illnesses^27^. Studies comparing FA profile between breeds support the use of Jersey cows, because of their improved FA profile, due to the higher percentage of nutritionally beneficial FA compared to HF cows^23^. However, results are inconsistent across studies and other works report higher concentrations of the nutritionally undesirable C16:0 and lower concentrations of the nutritionally beneficial OA in milk from Jersey and HF × J cows than HF cows^6,28^. Another study found no difference in milk C16:0 concentrations between HF and Jersey cows but higher C12:0 and C14:0 concentrations in Jersey compared to HF milk^29^.

Although some potential benefits of alternative breeds in low-input system have been investigated, there has not been a systematic analysis of the simultaneous effect of Jersey crossbreds on productivity, health, fertility parameters and milk nutritional quality. This study aims, for the first time, to simultaneously (1) quantify the effects of, and the interactions between, dairy cow genotypes (HF and HF × J) and season (spring, summer, autumn) on milk yield and basic composition; feed efficiency, health, and fertility parameters; and milk FA profiles, and (2) investigate associations between cow genotype (proportion of Jersey in the genetics : determined by breeding records from farmers’ as part of the questionnaire), and dietary drivers (type and amounts of pasture, conserved forage and concentrates) with milk yield and basic composition; efficiency, health, and fertility parameters; and milk FA profiles; using multivariate redundancy analyses (RDA).

## Materials and methods

All methods are reported in accordance with ARRIVE guidelines (https://arriveguidelines.org) for the reporting of animal experiments.

### Experimental design and collection of data and milk samples

The present study considers 219 milk samples collected from 73 cows, selected to represent the purebred HF and 50% HF:50% Jersey crossbred (HF × J), over 3 sampling periods (spring, summer, autumn) in four pasture-based low-input dairy farms, in England (Midlands and South) between March and October. The number of cows and their breeding groups within each farm are presented in the Supplementary Information (Table S1). All herds used both purebred HF and 50% HF: 50% Jersey crossbred (HF × J) cows; 3 block calving in spring and one in early autumn. Low-input dairy farming in the UK is generally characterised by high dietary contribution of pasture (> 80% DMI in the present study) during the grazing season and/or conserved forage, (mainly during winter housing); and low contribution of concentrate feeds (< 20% DMI in the present study)^3,30^. For all individual cows, a one-off questionnaire was used to collect data on pre-survey health, parity and most recent calving date as well as a breeding pedigree, based on farmers' records. A corresponding questionnaire, for each cow and milk sample, recorded milk yield, feeding practices (type/amounts of conserved forage, concentrates and supplements offered), disease incidences (mastitis, lameness, other) and fertility parameters (calving dates, calving to service interval, number of services to conception). Estimated DMI and pasture intake were calculated based on average breed live weight and recorded milk yield, as previously shown^3^. Live weights of cows were estimated based on mean weights of breeds (HF = 650 kg, Jersey = 450 kg) as previously recommended for low-input systems in the UK^3^. The estimated DMI and dietary components for the HF and HF × J cows in the current study are shown in Table 1. A detailed presentation of the same variables for each experimental farm is shown in the Supplementary Information (Table S1). Efficiency parameters were calculated as; (i) feed efficiency = milk yield (kg/d) / DMI (kg/d), (ii) feed non-grazing efficiency = milk yield (kg/d) / DMI (kg/d) excluding grazing, (iii) feed concentrate efficiency = milk yield (kg/d) / DMI (kg/d) from concentrate, (iv) fat efficiency = fat yield (g/d) / DMI (kg/d), (v) fat non-grazing efficiency = fat yield (g/d) / DMI (kg/d) excluding grazing, (vi) fat concentrate efficiency fat yield (g/d) / DMI (kg/d) from concentrate, (vii) protein efficiency = protein yield (g/d) / DMI (kg/d), (viii) protein non-grazing efficiency = protein yield (g/d) / DMI (kg/d) excluding grazing, and (ix) protein concentrate efficiency protein yield (g/d) / DMI (kg/d) from concentrate.

**Table 1:** Means ± SE and ANOVA P-values for the estimated dry matter (DM) intake (DMI) and dietary components (% of DMI) in 73 individual cows from two breeding groups (100% Holstein-Friesian, HF; 50% Holstein-Friesian:50% Jersey, HF × J) and different seasons in four low-input dairy farms in England and Wales.

	Breed				Season					Breed × Season
	HF	HF × J	SE	*P*-Value^2^	Spring	Summer	Autumn	SE	*P*-Value^2^	
	n^1^ = 96	n^1^ = 123			n^1^ = 59	n^1^ = 58	n^1^ = 59			*P*-value^2^
Days in Milk	169	164.5	10.72	0.939	147.6	176.1	177.6	12.81	0.191	0.387
Estimated Feed intake (kg DM/cow/day)	18.9	16.3	0.13	< 0.001	18.6^a^	17.2^b^	16.7^c^	0.21	< 0.001	0.804
**Diet components (% DMI unless otherwise stated)**										
Grazing	64.5	59.6	3.13	0.001	76.3^b^	81.1^a^	28.5^c^	2.19	< 0.001	0.546
Total forage	83.5	82.1	0.97	0.004	79.0^b^	90.8^a^	78.7^b^	0.92	< 0.001	0.596
Grass silage	8.9	12.3	1.53	0.251	2.5^c^	9.8^b^	19.9^a^	1.51	< 0.001	0.550
Maize silage	9.6	9.5	1.55	0.127	0.0^b^	0.0^b^	28.5^a^	0.41	< 0.001	0.095
Wholecrop	0.5	0.7	0.19	0.961	0.0^b^	0.0^b^	1.8^a^	0.12	< 0.001	0.122
Hay/Straw	0.1	0.1	0.05	0.719	0.2^a^	0.0^b^	0.0^b^	0.03	< 0.001	0.601
Moist by products	3.1	2.9	0.63	0.417	1.6^b^	0.0^b^	7.2^a^	0.48	< 0.001	0.591
Dry straight feeds	5.3	4.8	0.72	0.481	6.8^a^	2.8^c^	5.3^b^	0.80	< 0.001	0.304
Cereals	0.4	0.7	0.34	0.826	0.0^b^	0.0^b^	1.6^a^	0.24	< 0.001	0.663
Compound	7.7	9.6	1.12	0.071	12.6^a^	6.3^b^	7.2^b^	1.30	< 0.001	0.224
Minerals/Vitamins (g/cow/day)	55.0	53.7	5.72	0.885	41.1^b^	41.1^b^	80.6^a^	6.69	< 0.001	0.098

^1^ n is the number of records used to calculate means.

^2^ Significances were declared at *P* < 0.05. Means within a row, for season, with different upper-case letters are significantly different according to Fisher’s protected least significant difference test (*P* < 0.05).

### Milk analysis

Upon collection, milk samples were preserved with bronopol, one aliquot was sent directly to National Milk Laboratories, for commercial blinded analysis of basic composition (contents of milk fat, protein, urea, lactose) and somatic cell count (SCC), and another transferred to the laboratories of Newcastle University, where it was immediately stored and kept frozen at − 20 °C until FA profiling. Basic milk composition was analysed using Milkoscan FT 6000 (Foss Electric, Hillerød, Denmark), and SCC was recorded using a Fossomatic instrument (Foss Electric). Milk FA profile was blinded analysed using gas chromatography based on methodologies previously described by Stergiadis et al.^31^. Human health related indices (AI; atherogenicity index, TI; thrombogenicity index and HH ratio; hypocholesterolemic to hypercholesterolemic ratio), were calculated based on Srednicka-Tober et al.^32^ and Mierkita et al.^33^, respectively. Desaturase activity index was calculated based on Kay et al.^34^.

### Statistical analysis

A repeated measures, mixed linear model analysis was carried out (residual maximum likelihood analysis; REML), using Genstat® 18^35^ to investigate the effect of breed, month and their interaction on milk yield, basic composition, efficiency parameters and milk FA profiles. Experimental unit was the cow ID at a particular season. The fixed effects included breeding group (HF, HF × J), season (spring, summer, autumn; repeated measure) and their interaction, while individual cow ID (nested in Farm ID), were included as random effects. The effect of breed on health and fertility parameters in each cow (experimental unit) which were assessed as total across the year (cases of mastitis, lameness and other health; number of fertility treatments and services), were investigated using a generalised linear mixed model in Genstat® 18^35^using breed as fixed factor and Farm ID as random factor. When the fixed effect was significant (*p* < 0.05), pairwise comparisons of means were performed using Fisher’s Least Significant Difference test. Normality of the residuals were visually assessed and most showed no deviation from normality, except for fat non-grazing efficiency, somatic cell count, mastitis cases, all health cases, other health cases and services, which were log transformed prior to REML analysis. Descriptive statistics to generate means and standard errors for presentation in tables, were carried out in Minitab® 20.2.

**Figure 1:**
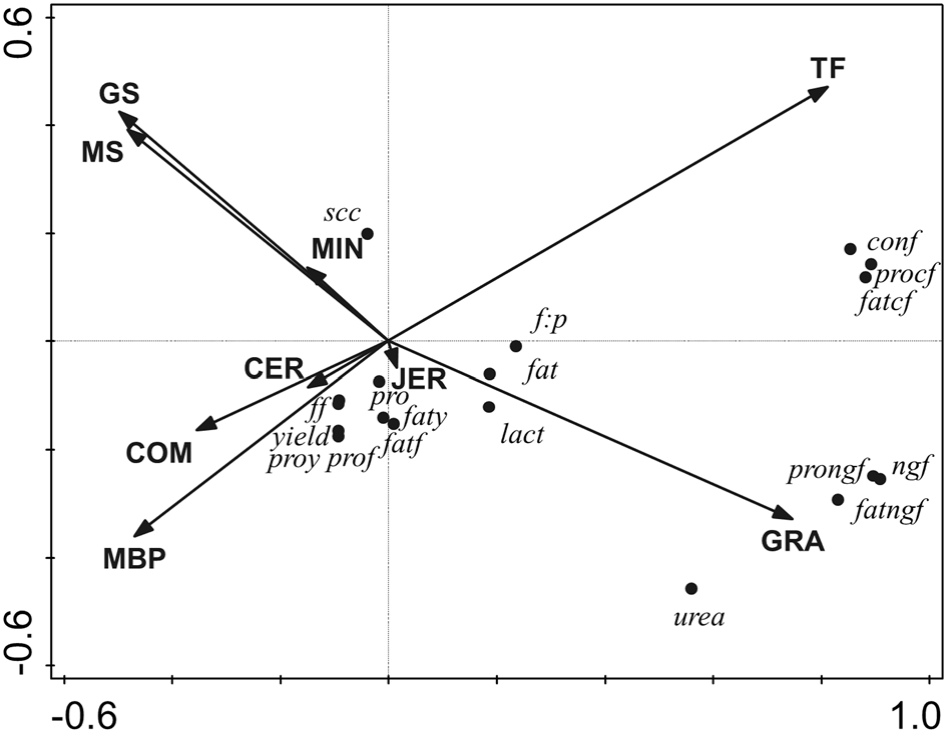
Biplot derived from the redundancy analysis, showing the relationship between diet composition parameters (total forage, TF; estimated grazing, GRA; grass silage, GS; maize silage, MS; hay/straw, HS; moist by-products, MBP; cereals, CER; minerals, MIN; compound feed, COM) and breed (Jersey, JER), relative to (i) milk yield (kg/cow/day), *(yield),* and basic composition parameters including milk fat yield (kg/cow/day), *(faty)*; milk protein yield (kg/cow/day), *(proy)*; milk fat content (g/kg milk), *(fat)*; milk protein content (g/kg milk), *(pro)*; fat:protein ratio, *(f:p)*; milk lactose content (g/kg milk), *(lact)*; milk urea content (g/kg milk), *(urea)*; milk SCC (× 1000/ml milk), *(scc)*; and (ii) efficiency parameters including feed efficiency (kg milk/kg DMI), *(ff)*; feed non-grazing efficiency (kg milk/kg non-grazing DMI), *(ngf)*; feed concentrate efficiency (kg milk/kg concentrate DMI), *(conf)*; fat efficiency (g fat yield/kg DMI), *(fatff)*; fat non-grazing efficiency (g fat yield/kg non-grazing DMI), *(fatngf)*; fat concentrate efficiency (g fat yield/kg concentrate DMI), *(fatcf)*; protein efficiency (g protein yield/kg DMI), *(fatff)*; protein non-grazing efficiency (g protein yield/kg DMI), *(prongf)*; protein concentrate efficiency (g protein yield/kg concentrate DMI), *(procf).* The total adjusted explained variation was 85.6%. Axis 1 explained 82.8% of the variation and Axis 2 explained a further 3.5% of the variation. Continuous variables, shown as arrows were the following (presented in order of contribution to the explained variation; *P*-value also shown in parentheses): TF (55.5%, *P* = 0.002), GRA (12.1%, *P* = 0.002), MS (5.5%, 0.002), COM (5%, *P* = 0.002), MBP (3.7%, *P* = 0.002), MIN (3.1%, *P* = 0.002), GS (1.2%, *P* = 0.001), JER (6.6%, *P* = 0.0.006), CER (< 0.1%, *P* = 0.518).

**Figure 2:**
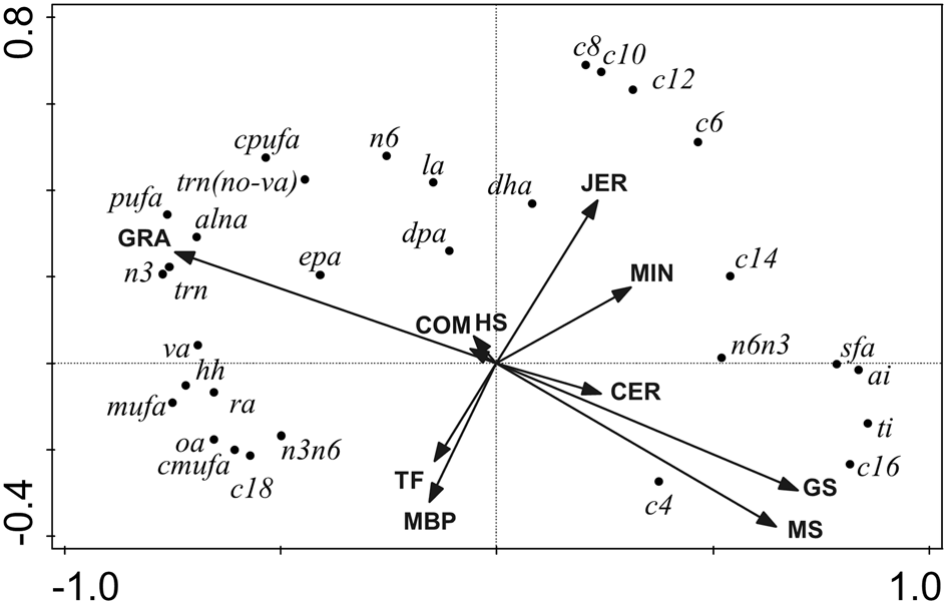
Biplot derived from the redundancy analysis, showing the relationship between diet composition parameters (total forage, TF; estimated grazing, GRA; grass silage, GS; maize silage, MS; hay/straw, HS; moist by-products, MBP; cereals, CER; minerals, MIN; compound feed, COM) and breed (Jersey, JER), relative to milk concentrations of butyric acid (c4), caproic acid (c6), caprylic acid (c8), capric acid (c10), lauric acid (c12), myristic acid (c14), palmitic acid (c16), stearic acid (c18), vaccenic acid (va), oleic acid (oa), linoleic acid (la), rumenic acid (ra), α-linolenic acid (alna), eicosapentaenoic acid (epa), docosapentaenoic acid (dpa), docosahexaenoic acid (dha), saturated fatty acids (sfa), monounsaturated fatty acids (mufa), cis-monounsaturated fatty acids (cmufa), polyunsaturated fatty acids (pufa), cis-polyunsaturated fatty acids (cpufa), omega-3 fatty acids (n3), omega-6 fatty acids (n6), omega-3:omega-6 (n3n6), omega-6:omega-3 (n6n3), trans fatty acids (trn), trans fatty acids excluding vaccenic acid (trn(no-va)), atherogenicity index (ai), thrombogenicity index (ti) and hypocolesterolemic to hypercolesterolemic ratio (hh). The total adjusted explained variation was 57.7%. Axis 1 explained 54.2% of the variation and Axis 2 explained a further 3.3% of the variation. Continuous variables, shown as arrows were the following (presented in order of contribution to the explained variation; *P*-value also shown in parentheses): GRA (30.3%, *P* = 0.002), COM (10.0%, *P* = 0.002), MBP (10.0%, *P* = 0.002), TF (3.2%, *P* = 0.002), JER (2.5%, *P* = 0.002), CER (1.0%, *P* = 0.022), MIN (1.5%, *P* = 0.018), HS (0.9%, *P* = 0.04), GS (0.4%, *P* = 0.158), MS (0.3%, *P* = 0.242).

**Figure 3:**
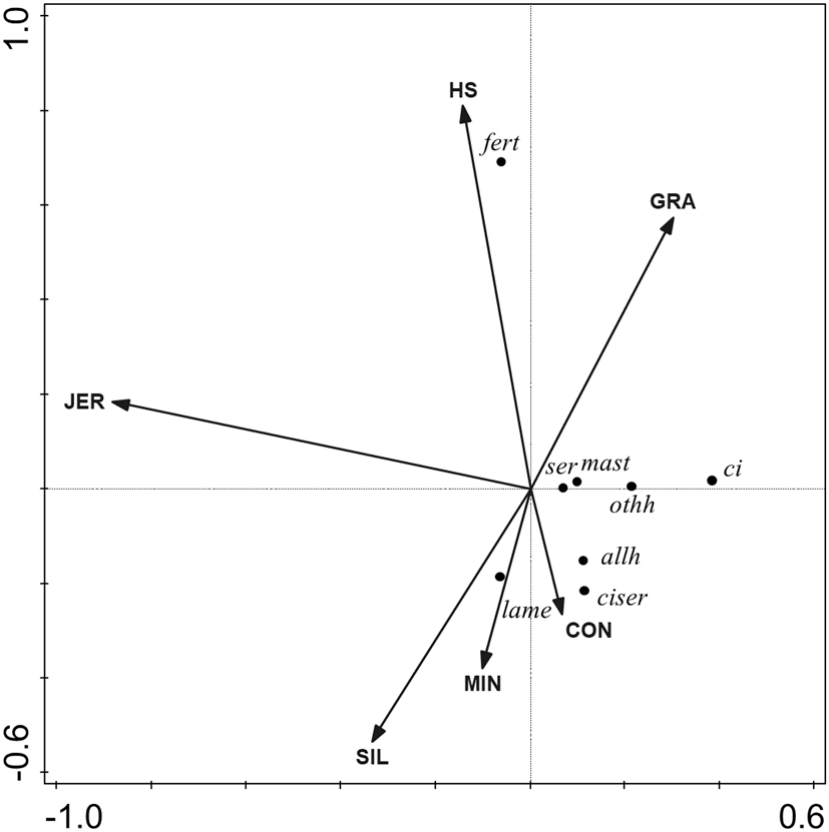
Biplot derived from the redundancy analysis, showing the relationship between diet composition parameters (estimated grazing, GRA; silages (grass silage, maise silage, whole crop silage), SIL; hay/straw, HS; concentrates (moist by-products, cereals, compound feed, dry straights), CON; minerals, MIN) and breed (Jersey, JER), relative to mastitis cases (mast), lameness (lame), other health cases (othh), all health cases (allh), fertility (fert), calving interval (ci), services (ser), and calving to first service interval (cfs). The total adjusted explained variation was 14.9%. Axis 1 explained 14.0% of the variation and Axis 2 explained a further 0.9% of the variation. Continuous variables, shown as arrows were (presented in order of contribution to the explained variation and P-value in parentheses): JER (10.9%, *P* = 0.008), HS (1.6%, *P* = 0.298), MIN (1.4%, *P* = 0.314), SIL (0.9%. *P* = 0.482), GRA (0.2%, *P* = 0.834), CON (0.1%, *P* = 0.914).

Multivariate redundancy analysis (RDA) was carried out using Canoco5®^36^ to further investigate the impact of breed and diet on productivity, milk basic composition and efficiency parameters, FA profiles and health and fertility parameters. In the RDA biplots for productivity, basic composition, and efficiency parameters, and FA profiles, arrow length and direction represent the relative effects of driver variables (breed and diet composition parameters) on the response variables (productivity, basic composition, efficiency parameters, FA profiles). The driver related to breed represents the contribution of Jersey genetics to the genome. Drivers related to nutrition were dietary proportions of estimated grazing (GRA), total forage (TF), grass silage (GS), Maize silage (MS), wholecrop silage (WC), hay/straw (HS), moist by-products (MBP), dry straights (DRY), cereals (CER), compound (COM) and minerals and vitamins (MIN). The response variables in Fig. 1 included milk yields of fat and protein; fat:protein ratio; milk contents of fat content, protein, lactose, and urea; SCC; efficiency parameters (feed, non-grazing, concentrate, fat, fat non-grazing, fat concentrate, protein, protein non-grazing, protein concentrate). The response variables in Fig. 2 included milk FA profile (butyric acid, C4:0; caproic acid, C6:0; caprylic acid, C8:0; capric acid, C10:0; lauric acid, C12:0; myristic acid, C14:0; palmitic acid, C16:0; stearic acid, C18:0; vaccenic acid, VA; oleic acid, OA; linoleic acid, LA; rumenic acid, RA; α-linolenic acid, ALNA; eicosapentaenoic acid, EPA; docosapentaenoic acid, DPA; docosahexaenoic acid, DHA; saturated FA, SFA; monounsaturated FA, MUFA; *cis-*monounsaturated FA, cMUFA; polyunsaturated FA, PUFA; *cis*-polyunsaturated FA, cPUFA; omega-3 polyunsaturated FA, n-3; omega-6 polyunsaturated FA, n-6; n-3:n-6 ratio; n-6:n-3 ratio; *trans* FA; *trans* FA excluding VA; Atherogenicity index, AI; Thrombogenicity index, TI; Hypocholesterolemic to Hypercholesterolemic ratio, HH). In the RDA biplot for health and fertility parameters (Fig. 3), the arrow length and direction represent the relative effects of driver variables (breed and diet composition parameters) on the response variables (health and fertility). The driver related to breed represents the contribution of Jersey genetics to the pedigree. Drivers related to nutrition were dietary proportions of estimated grazing, GRA; silages (grass silage, maize silage and whole crop silage), SIL; hay/straw, HS; concentrates (moist by-products, cereals, compound feed and dry straights), CON; minerals, MIN. The response variables included total incidences of mastitis, lameness, other health cases and all health cases, and fertility, calving interval, services, and calving interval:service ratio.

### Ethical approval

The animal study was reviewed and approved by all procedures were acceptable to internal ethical review, in accordance with EU Directive 2010/63/EU for animal experiments and approved by the Animal Welfare and Ethical Review Body at Newcastle University. Written informed consent was obtained from the owners for the participation of their animals in this study.

## Results

All differences presented here (and subsequently discussed) are statistically significant (*P* < 0.05) unless otherwise stated.

### Animal diets

In comparison with HF cows, HF × J cows had lower estimated DMI (-2.6 kg/day), and lower grazing and total forage intakes (−4.91% and −1.39% DMI, respectively) than HF cows but all other individual dietary components did not differ. Significant effects of season were identified for estimated DMI and all individual diet components (Table 1). Estimated DMI decreased from spring to summer (− 1.4 kg DM/cow/day) and from summer to autumn (− 0.5 kg DM/cow/day). Grazing contribution in cows’ diets was highest in summer, intermediate in spring and lowest in autumn; with an overall decrease between summer and autumn of − 47.8% DMI. Total forage intake was also highest in summer compared to spring and autumn (+ 11.8% and + 12.1% DMI respectively). Intakes of grass silage increased from spring to summer by + 7.3% DMI and a further 10.1% DMI between summer and autumn. Intakes of maize silage, wholecrop, moist by-products, cereals and minerals were higher in autumn than in spring (+ 28.5%, + 1.8%, + 5.6%, + 1.6% DMI, and + 39.5 g/cow/day, respectively) and summer (+ 28.5%, + 1.8%, + 7.2%, + 1.6% DMI, and + 39.5 g/cow/day respectively). Intakes for hay and straw and compound feed were higher in spring than in summer (+ 0.2% and + 6.3% DMI respectively) and autumn (+ 0.2% and + 5.4% DMI respectively). Dry straights intake decreased from spring to summer (− 4.0% DMI) and increased from summer to autumn (+ 2.5% DMI).

### Milk production, basic composition and efficiency

#### Effect of breed and season

Significant effect of breed was identified for milk fat, protein and casein concentrations, and efficiencies of feed, fat and protein (Table 2). HF × J cows produced milk with higher concentrations of fat (+ 3.2 g/kg milk), protein (+ 2.9 g/kg milk) and casein (+ 2.7 g/kg milk) than HF cows. Feed, fat and protein efficiencies were higher in HF × J cows than HF, by + 0.12 kg milk, + 7.1 g fat, + 7.3 g protein, for every kg of DMI.

**Table 2:** Means ± SE and *P*-values for breed and season on the yield, basic composition and efficiency parameters of milk collected from 73 individual cows from two breeding groups (100% Holstein-Friesian, HF; 50% Holstein-Friesian:50% Jersey, HF × J) and different seasons in four low-input dairy farms in England and Wales.

	Breed				Season					Breed × Season
	HF	HF × J	SE	*P*-Value^2^	Spring	Summer	Autumn	SE	*P*-Value^2^	
	n^1^ = 96	n^1^ = 123			n^1^ = 59	n^1^ = 58	n^1^ = 59			*P*-Value^2^
**Productivity (kg/cow/day)**										
Milk yield	21.3	20.5	1.02	0.252	29.3^a^	19.2^b^	14.1^c^	0.93	< 0.001	0.804
Milk fat yield	0.81	0.81	0.041	0.763	1.17^a^	0.69^b^	0.56^c^	0.034	< 0.001	0.900
Milk protein yield	0.72	0.74	0.033	0.441	1.02^a^	0.63^b^	0.52^c^	0.028	< 0.001	0.613
**Basic composition (g/kg milk)**										
Milk fat	38.6	41.8	1.25	0.033	41.6	38.7	40.6	1.54	0.274	0.959
Milk protein	34.6	37.5	0.64	< 0.001	35.3^b^	34.2^b^	38.9^a^	0.71	< 0.001	0.841
Milk casein	25.6	28.3	1.08	< 0.001	-	24.0	27.9	0.87	< 0.001	0.038
Milk whey protein	8.80	8.31	0.440	0.577	-	7.86	8.83	0.346	0.194	0.693
Fat:protein (g/g)	1.12	1.12	0.029	0.970	1.17	1.12	1.05	0.035	0.061	0.916
Milk lactose (g/kg milk)	44.1	45.4	0.57	0.468	48.1^a^	45.0^b^	42.8^c^	0.64	< 0.001	0.863
Urea (g/l milk)	0.17	0.18	0.020	0.235	0.25^a^	0.21^b^	0.10^c^	0.016	< 0.001	0.235
Milk SCC (× 1000/ml milk)	175.3	173.6	50.78	0.911	144.7	204.4	167.6	57.51	0.802	0.386
**Efficiency parameters**										
Feed efficiency (kg milk/kg DMI)	1.10	1.22	0.048	0.027	1.56^a^	1.10^b^	0.83^c^	0.045	< 0.001	0.474
Feed non-grazing efficiency ( kg milk/kg non-grazing DMI)	7.74	6.96	1.029	0.310	10.4^b^	10.8^a^	1.16^c^	0.896	< 0.001	0.907
Feed concentrate efficiency (kg milk/kg concentrate DMI)	8.46	7.95	0.581	0.066	7.12^b^	13.1^a^	4.80^c^	0.506	< 0.001	0.836
Fat efficiency (g fat/kg DMI)	41.9	49.0	2.04	< 0.001	62.8^a^	40.5^b^	33.1^c^	1.86	< 0.001	0.512
Fat non-grazing efficiency (g fat/kg non-grazing DMI)	310.5	311.4	55.89	0.799	473.1^a^	446.7^a^	47.7^b^	49.22	< 0.001	0.939
Fat concentrate efficiency (g fat/kg concentrate DMI)	315.2	316.8	26.73	0.474	279.8^b^	527.6^a^	191.6^c^	24.43	< 0.001	0.831
Protein efficiency (g protein/kg DMI)	37.0	44.3	1.55	< 0.001	54.5^a^	36.9^b^	30.9^c^	1.41	< 0.001	0.206
Protein non-grazing efficiency (g protein/kg non-grazing DMI)	256.4	251.6	37.90	0.604	361.8^b^	389.3^a^	44.8^c^	33.34	< 0.001	0.661
Protein concentrate efficiency (g protein/kg concentrate DMI)	282.1	286.1	21.56	0.328	248.5^b^	471.5^a^	177.3^c^	18.85	< 0.001	0.595

^1^ n is the number of records used to calculate means ± SE and *P*-values. Data for milk casein and whey protein were not collected for spring.

^2^ Significances were declared at *P* < 0.05. Means within a row, for season, with different upper-case letters are significantly different according to Fisher’s protected least significant difference test (*P* < 0.05).

#### Effect of season

Significant effects of season were identified for all productivity and efficiency parameters and milk concentrations of protein, casein, lactose and urea (Table 2). Yields and efficiencies of milk, fat and protein were highest in spring, intermediate in summer and lowest in autumn; from spring to autumn (i) yields were decreased by − 15.2 kg for milk, − 0.61 kg for fat and − 0.50 kg for protein, and (ii) cows produced − 0.73 kg less milk, − 29.7 g less fat and − 23.6 g less protein, per kg of DMI. Milk protein in autumn was higher than in spring (+ 3.6 g/kg milk) and summer (+ 4.7 g/kg milk). When compared with autumn, milk lactose and urea concentrations were higher in spring, intermediate in summer and lowest in autumn and ranged by 5.3 g/kg milk for lactose and 0.15 g/kg for urea. Feed and protein non-grazing efficiency was highest in summer, intermediate in spring and lowest in autumn and ranged by 9.64 kg milk for feed and 344.5 g for protein. When compared with autumn, cows in spring and summer produced more fat (+ 425.4 g and + 399.0 g, respectively) per kg of non-grazing DMI. Concentrate efficiencies of milk, fat and protein were highest in summer, intermediate in spring and lowest in autumn; between the seasons with maximum (summer) and minimum (autumn) concentrate efficiencies, the production per kg concentrate DMI, ranged by 8.3 kg for milk, 336.0 g for fat and 294.2 g for protein.

#### *Effect of the breed* × *season interaction*

Significant effects of the breed × season interaction (Table S3) were identified for milk casein contents, but there were no records for spring. In autumn, HF × J cows produced milk with + 4.3 g/kg milk more casein (30.3 g/kg milk) than HF cows (26.0 g/kg milk); but no difference was observed in summer. The effect of breed × season interaction was not significant (*P* > 0.05) for the other production, basic composition and efficiency parameters.

#### Multivariate analyses of the effect of Jersey genetics and diet composition on milk basic composition

The RDA biplot showing the relative impact of feed and breed drivers on milk yield, composition and efficiency parameters is demonstrated in Fig. 1. Drivers together explained 86.6% of the variation, of which 82.8% was explained by axis 1 and a further 3.5% was explained by axis 2. Total forage and grazing intakes accounted for 55.5% and 12.1% of the variation, respectively. Maize silage, compound feed intake and Jersey genetics explained 5.5%, 5.0% and 6.6% respectively, while other individual feeds explained < 5% of the variation each. Intakes of grazing and total forage were positively correlated with protein concentrate efficiency, fat concentrate efficiency, concentrate efficiency, protein non-grazing efficiency, non-grazing efficiency, fat non-grazing efficiency and milk urea concentrations, as well as, (to a lesser extent) milk content of fat, lactose and fat:protein. The same response variables were negatively associated with intakes of grass silage, maize silage, moist by-products and compound feed, and at a lesser extent, intake of cereals and minerals. Milk SCC was positively associated with grass silage, maize silage and mineral intakes and negatively correlated with grazing intake. Protein yield, efficiency and content in milk, fat efficiency and yield, milk yield, and feed efficiency were positively correlated with intakes of moist by-products, compound feed, and cereals and negatively correlated with total forage intake.

### Milk fatty acid profile

#### Effect of breed

Significant effects for breed were found for all individual SFA and overall SFA, excluding C16:0 (Table 3). HF × J cows produced milk with higher concentration of overall SFA (+ 27.3 g/kg FA), and individual SFA; C4:0 (+ 2.6 g/kg FA), C6:0 (+ 1.9 g/kg FA), C8:0 (+ 1.3 g/kg FA), C10:0 (+ 3.0 g/kg FA), C12:0 (+ 3.7 g/kg FA) and C14:0 (+ 4.6 g/kg FA) than HF. HF cows produced higher milk concentrations of total MUFA (+ 23.7 g/kg FA), OA (+ 17.3 g/kg FA), *cis* MUFA (+ 22.3 g/kg FA), PUFA (+ 3.6 g/kg FA), *trans* PUFA (+ 0.04 g/kg FA), *cis/trans* + *trans cis* PUFA (+ 0.25 g/kg FA) and total *trans* FA (excluding VA) (+ 0.22 g/kg FA) than HF × J cows. n−3:n-6 ratio was higher for HF × J cows compared to HF cows. AI and TI were higher in HF × J than HF milk. HF cows had a higher HH ratio and higher desaturase activity index (Δ^9^I; desaturase activity index, C14:0/C14:1, C16:1/C16:0, OA/C18:0 and RA/VA) than HF × J cows. The effect of breed on all measured individual FAs is presented in the Supplementary Information (Table S2).

**Table 3:** Means ± SE and *P*-values for breed and season on the fatty acid profile of milk collected from 73 individual cows from two breeding groups (100% Holstein-Friesian, HF; 50% Holstein-Friesian:50% Jersey, HF × J) and different seasons in four low-input dairy farms in England and Wales.

	Breed				Season					Breed × Season
	HF	HF × J	SE	*P*-value^2^	Spring	Summer	Autumn	SE	*P*-value^2^	
	n^1^ = 83	n^1^ = 95			n^1^ = 58	n^1^ = 66	n^1^ = 54			*P*-value^2^
**Individual FA (g/kg total FA)**										
*SFA*										
C4:0	27.2	29.8	0.66	0.015	24.3^b^	31.3^a^	29.9^a^	0.73	< 0.001	0.285
C6:0	25.7	27.6	0.44	0.001	27.9^a^	25.6^b^	27.0^a^	0.53	< 0.001	0.217
C8:0	13.6	14.9	0.29	< 0.001	16.0^a^	13.5^b^	13.4^b^	0.33	< 0.001	0.398
C10:0	30.4	33.4	0.83	0.002	36.9^a^	29.3^b^	30.0^b^	0.93	< 0.001	0.546
C12:0	36.4	40.1	1.00	< 0.001	45.7^a^	31.6^c^	38.7^b^	1.02	< 0.001	0.515
C14:0	115.0	119.6	1.57	0.011	117.1^b^	112.4^c^	124.0^a^	1.83	< 0.001	0.821
C16:0	306.8	311.8	5.71	0.356	276.0^c^	292.1^b^	366.7^a^	4.71	< 0.001	0.750
C18:0	94.0	100.2	2.55	0.186	94.1^b^	112.2^a^	82.6^c^	2.68	< 0.001	0.375
*MUFA*										
VA	22.9	23.3	1.51	0.962	27.3^a^	29.4^a^	11.0^b^	1.50	< 0.001	0.313
OA	191.9	174.6	4.18	0.001	187.9^a^	197.1^a^	159.3^b^	4.78	< 0.001	0.887
*PUFA*										
LA	10.1	9.55	0.421	0.233	13.2^a^	7.15^c^	9.38^b^	0.381	< 0.001	0.301
RA	11.3	10.1	0.70	0.150	11.9^a^	13.2^a^	6.26^b^	0.762	< 0.001	0.576
ALNA	6.95	7.01	0.256	0.992	8.51^a^	7.63^b^	4.56^c^	0.224	< 0.001	0.608
EPA	0.80	0.79	0.026	0.493	0.89^a^	0.87^a^	0.61^b^	0.026	< 0.001	0.611
DPA	1.15	1.12	0.035	0.857	1.18^a^	1.18^a^	1.01^b^	0.041	0.003	0.813
DHA	0.08	0.09	0.006	0.249	0.10^a^	0.09^b^	0.07^b^	0.008	0.003	0.233
**FA groups (g/kg total FA)**										
SFA^3^	675.0	702.3	6.30	< 0.001	663.7^b^	673.3^b^	737.2^a^	6.71	< 0.001	0.674
MUFA^4^	277.2	253.5	5.22	< 0.001	279.5^a^	279.8^a^	229.7^b^	5.83	< 0.001	0.681
*cis* MUFA^6^	235.4	213.1	4.18	< 0.001	228.4^a^	234.9^a^	204.3^b^	5.02	< 0.001	0.766
*trans* MUFA^7^	41.8	40.4	1.83	0.454	51.1^a^	44.9^b^	25.4^c^	1.77	< 0.001	0.454
PUFA^5^	47.9	44.3	1.42	0.021	56.8^a^	47.0^b^	33.1^c^	1.26	< 0.001	0.586
*cis* PUFA^8^	23.7	23.0	0.61	0.273	28.9^a^	21.7^b^	19.2^c^	0.53	< 0.001	0.639
*trans* PUFA^9^	3.55	3.20	0.123	0.022	4.27^a^	3.33^b^	2.43^c^	0.119	< 0.001	0.479
*cis/trans* + *trans/cis* PUFA^10^	20.6	18.1	0.95	0.039	23.65^a^	21.92^a^	11.39^b^	0.952	< 0.001	0.731
n-3^11^	13.9	13.9	0.49	0.996	16.1^a^	16.0^a^	8.92^b^	0.41	< 0.001	0.708
n-6^12^	15.4	14.2	0.52	0.053	19.4^a^	11.6^c^	13.5^b^	0.45	< 0.001	0.322
n-3/n-6 ratio	0.99	1.06	0.050	0.048	0.90^b^	1.42^a^	0.68^c^	0.040	< 0.001	0.727
n-6/n-3 ratio	1.22	1.14	0.060	0.230	1.22^b^	0.76^c^	1.64^a^	0.048	< 0.001	0.327
*trans* FA^13^	45.3	43.6	1.91	0.400	55.4^a^	48.3^b^	27.9^c^	1.82	< 0.001	0.504
*trans* FA (exc. VA)	22.4	20.2	0.77	0.028	28.1^a^	18.9^b^	16.8^b^	0.68	< 0.001	0.762
**Human health related indices**										
AI^14^	2.62	2.98	0.10	0.002	2.41^b^	2.47^b^	3.68^a^	0.097	< 0.001	0.135
TI^15^	2.77	3.06	0.10	0.004	2.39^b^	2.56^b^	3.92^a^	0.085	< 0.001	0.230
HH^16^	0.55	0.49	0.02	0.003	0.57^a^	0.58^a^	0.38^b^	0.020	< 0.001	0.992
**Δ**^**9**^**-desaturase activity indicators**										
Δ^9^I^17^	3.00	2.76	0.005	< 0.001	3.04^a^	3.02^a^	2.54^b^	0.006	< 0.001	0.786
C14:1/C14:0	0.09	0.08	0.003	0.002	0.08^b^	0.08^b^	0.10^a^	0.003	< 0.001	0.450
C16:1/C16:0	0.07	0.06	0.001	< 0.001	0.06	0.06	0.06	0.002	0.202	0.567
OA/C18:0	2.16	1.79	0.054	< 0.001	2.07^a^	1.77^b^	2.09^a^	0.066	< 0.001	0.171
RA/VA	0.53	0.44	0.015	< 0.001	0.45^b^	0.45^b^	0.57^a^	0.017	< 0.001	0.032

^1^ n is the number of records used to calculate means.

^2^ Significances were declared at *P* < 0.05. Means within a row, for season, with different upper-case letters are significantly different according to Fisher’s protected least significant difference test (*P* < 0.05).

^3^SFA: C4:0, C5:0, C6:0, C7:0, C8:0, C9:0, C10:0, C11:0, C12:0, C13:0, C14:0, C15:0, C16:0, C17:0, C18:0, C20:0, C22:0, C24:0.

^4^MUFA: c9 C14:1, c9 C15:1, t9 C16:1, c9 C16:1, c9 C17:1, t6 + t7 + t8 C18:1, t9 C18:1, t10 C18:1, t11 C18:1, t12 + t13 + t14 C18:1, c9 C18:1 (OA), t15 C18:1, c11 C18:1, c12 C18:1, c13 C18:1, c14 + t16 C18:1, c15 C18:1, c15 C18:1, c10 C19:1, c5 C20:1, c8 C20:1, c13 C22:1, c15 C24:1.

^5^PUFA: t11t15 C18:2, t11t15 C18:2, t10t14 C18:2. C9t13 C18:2, t9t12 C18:2, t8c13 C18:2, c9t12 C18:2, t9c12 C18:2, t11c15 C18:2, c9c12 C18:2 (LA), two unidentified C18:2, c9c15 C18:2, c12c15 C18:2, c6c9c12 C18:3 (GLA), c9c12c15 C18:3 (ALN), c9t11 18:2 (RA), four unidentified conjugated linoleic acid (CLA) isomers, t11c13 C18:2 CLA, c9c13c15 C18:3, c11c14 C20:2, c9c11c15 C18:3, c8c11c14 C20:3, c11c14c17 C20:3, c5c8c11c14 C20:4, c13c16 C22:2, c5c8c14c17 C20:5 (EPA), c13c16c19 C22:3, c7c10c13c16 C22:4, c7c10c13c16c19 C22:5 (DPA), c7c10c13c16c19 C22:6 (DHA).

^*6*^*cis* MUFA: c9 C14:1, c9 C15:1, c9 C16:1, c9 C17:1, c9 C18:1 (OA), c11 C18:1, c12 C18:1, c13 C18:1, c14 C18:1, c15 C18:1, c10 C19:1, c5 C20:1, c8 C20:1, c13 C22:1, c15 C24:1.

^*7*^*trans* MUFA: t9 C16:1, t6 + t7 + t8 C18:1, t9 C18:1, t10 C18:1, t11 C18:1 (VA), t12 + t13 + t14 C18:1, t15 C18:1, t16 C18:1.

^8^*cis*PUFA: c9c12 C18:2 (LA), unidentified *cis/cis* C18:2, c9c15 C18:2, c12c15 C18:2, c6c9c12 C18:3 (GLA), c9c12c15 C18:3 (ALN), c9c13c15 C18:3, c11c14 C20:2, c9c11c15 C18:3, c8c11c14 C20:3, c11c14c17 C20:3, c5c8c11c14 C20:4, c13c16 C22:2, c5c8c11c14c17 C20:5 (EPA), c13c16c19 C22:3, c7c10c13c16 C22:4, c7c10c13c16c19 C22:5 (DPA), c7c10c13c16c19 C22:6 (DHA).

^9^*trans* PUFA:t11t15 C18:2, t10t14 C18:2, t9t12 C18:2, unidentified *trans/trans* C18:2, unidentified *trans/trans* CLA isomers.

^10^*cis/trans* + *trans/cis* PUFA: c9t13 C18:2, t8c13 C18:2, c9t12 C18:2, t9c12 C18:2, ct1014 + 1216 C18:2, t11c15 C18:2, unidentified *cis/trans* + *trans/cis* C18:2, RA, t11c13 CLA, unidentified *cis/trans* + *trans/cis* CLA.

^11^Omega-3 PUFA (n-3): t11t15 C18:2, t11c15 C18:2, c9c15 C18:2, c12c15 C18:2, c9c12c15 C18:3 (ALN), c9c13c15 C18:3, c9c11c15 C18:3, c11c14c17 C20:3, c5c8c11c14c17 C20:5 (EPA), c13c16c19 C22:3, c7c10c13c16c19 C22:5 (DPA), c7c10c13c16c19 C22:6 (DHA).

^12^Omega-6 PUFA(n-6): t9t12 C18:2, c9t12 C18:2, t9c12 C18:2, c9c12 C18:2 (LA), c6c9c12 C18:3 (GLA), c11c14 C20:2, c8c11c14 C20:3, c5c8c11c14 C20:4, c13c16 C22:2, c7c10c13c16 C22:4.

^13^*trans* FA: t9 C16:1, t6 + t7 + t8 C18:1, t9 C18:1, t10 C18:1, t11 C18:1 (VA), t12 + t13 + t14 C18:1, t15 C18:1, c14 + t16 C18:1. t9 C16:1, t6 + t7 + t8 C18:1, t9 C18:1, t10 C18:1, t11 C18:1 (VA), t12 + t13 + t14 C18:1, t15 C18:1, t11t15 C18:2, t10t14 C18:2, t9t12 C18:2, t11c15 C18:2, t11t15 C18:2, t10t14 C18:2, t9t12 C18:2, unidentified trans/trans C18:2, unidentified trans/trans CLA.

^14^Atherogenicity index = (C12:0 + 4 × C14:0 + C16:0)/(MUFA + PUFA), as described in Srednicka-Tober et al. ^32^.

^15^Thrombogenicity index = (C14:0 + C16:0 + C18:0)/[(0.5 × MUFA) + (0.5 × n-6) + (3 × n-3) + (n-3/n-6)] as described in Srednicka-Tober et al. ^32^.

^16^Hypocholesterolemic to hypercholesteremic ratio = (C18:1 *cis*9 + total PUFA) / (C12:0 + C14:0 + C16:0).

^17^Δ9-desaturase activity index = (c9 C14:1 + c9 C16:1 + OA + RA)/(c9C14:1 + c9 C16:1 + OA + RA + C14:0 + C16:0 + C18:0 + VA) as described in Kay et al.^34^.

#### Effect of season

Significant differences were observed between seasons for all individual SFA, MUFA and PUFA (*P* < 0.05) (Table 3). Milk had a higher concentration of C4:0 in summer (+ 7.0 g/kg) and autumn (+ 5.6 g/kg), than in spring. Milk had a higher concentration of C6:0 in spring (+ 2.3 g/kg) and autumn (+ 1.4 g/kg) than in summer. Concentrations of C8:0, C10:0, DHA and total *trans* FA without VA were higher for spring than summer and autumn; between the seasons with maximum (spring) and minimum (summer/autumn) values, concentration ranged by 2.6 g/kg FA for C8:0, 7.6 g/kg FA for C10:0, 0.03 g/kg FA for DHA and 11.3 g/kg for total *trans* FA excluding VA. Concentration of C12:0, LA and n-6 were higher in spring, intermediate in autumn and lowest in summer; the difference between the highest value (spring) and the lowest value (summer) was + 14.1 g/kg FA for C12:0, 6.1 g/kg FA for LA and + 7.8 g/kg FA for n-6. Concentrations of C14:0 and the ratio of n−6:n−3 were highest in autumn, intermediate in spring and highest in summer; C14:0 was − 6.9 g/kg FA lower in spring than in autumn and -11.6 g/kg FA lower in summer than in autumn. C16:0 concentrations increased significantly from spring to autumn; cows produced- 90.7 g/kg FA and − 74.6 g/kg FA less in spring and summer, respectively, than in autumn. Milk concentrations of C18:0, and milk n−3:n−6 ratio were highest in summer, intermediate in spring and lowest in autumn. In summer, cows produced milk with + 29.9 g/kg FA and + 18.1 g/kg FA more C18:0 than autumn and spring, respectively. The individual FAs VA, OA, RA, EPA, DPA and the FA groups MUFA, *cis* MUFA, *cis/trans* + *trans/cis* PUFA, n−3 and Δ^9^I were higher in spring and summer than in autumn. Differences between the highest values (spring/summer) and lowest value (autumn) were 18.4 g/kg FA for VA, 37.8 g/kg FA for OA, 7.0 g/kg FA for RA, 0.28 g/kg FA for EPA, 0.17 g/kg FA for DPA, 50.1 g/kg FA for total MUFA, 30.6 g/kg FA for *cis* MUFA, 12.3 g/kg FA for *cis/trans* + *trans/cis* PUFA, and 7.2 g/kg FA for n-3. Milk concentrations of ALNA, *trans* MUFA, total PUFA, *cis* PUFA, *trans* PUFA and *trans* FA decreased from spring to autumn. Concentrations were decreased by − 3.95 g/kg FA for ALNA, − 25.7 g/kg FA for *trans* MUFA, − 23.7 g/kg FA for PUFA, − 9.7 g/kg FA for *cis* PUFA, − 1.84 g/kg FA for *trans* PUFA and 27.5 g/kg FA for *trans* FA. Total SFA, human health related indices (AI, TI) and RA/VA ratio were higher in autumn than in spring and summer. SFA was higher in autumn than in spring and summer, by + 73.5 g/kg FA and + 63.9 g/kg FA, respectively. The OA/C18:0 ratio was lower in summer than in spring and autumn.

#### *Effect of the breed* × *season interaction*

Significant effects of the breed × season interaction for FA profile (Table S4) were found for RA/VA; HF cows had higher concentrations of RA/VA in spring and autumn than HF × J cows.

#### Multivariate analyses of the effect of Jersey genetics and diet composition on FA profile

The RDA biplot showing the relative impact of feed and breed drivers on milk FA profile is demonstrated in Fig. 2. Drivers explained 57.7% of the total variation, of which 54.2% was explained by axis 1 and a further 3.3% was explained by axis 2. Grazing intake accounted for 30.3% of the variation and compound feed and moist-by-products explained a further 20.0% of the variation (10.0% each). Total forage intake, Jersey genetics and cereals intake explained 3.2%, 2.5% and 1%, respectively, while mineral/vitamin supplements intake explained a further 1.5%. All other diet components accounted for < 1% of the variation each. Jersey genetics were positively associated with milk concentrations of C6:0, C8:0, C10:0, C12:0, C14:0, LA, DPA, DHA, n-6 and n-6:n-3. Mineral intake was positively associated with milk concentrations of C6:0, C8:0, C10:0, C12:0, C14:0, n-6:n-3 and AI. Grass silage, maize silage and to a lesser extent, cereal intake were positively associated with C4:0, C14:0, C16:0, SFA, n-6:n-3 milk concentrations as well as human health indices (AI, TI). Total forage and moist by product intakes were positively associated with C18:0, HH, VA, OA, RA, MUFA, cMUFA, n-3:n-6 and negatively associated with C6:0, C8:0, C10:0, C12:0, C14:0, LA, DPA, DHA, SFA, n-6, n-6:n-3 and AI. Grazing intake was positively associated with C18:0, HH, VA, OA, PUFA, LA, RA, ALNA, EPA, DPA, MUFA, cPUFA, n-3, n-6, n-3:n-6, *trans*FA, *trans*FA without VA.

### Health and fertility

#### Effect of breed

Significant effects of breed were identified for calving interval with HF × J cows 48.3 days shorter than HF cows. The effect of breed was not statistically significant (*P* > 0.05) for other health and fertility parameters (Table 4). However, numerical values for total health cases and individual diseases were higher than in HF than HF × J cows, and there was a tendency for a significant effect for total health cases per cow per year. Therefore, future studies in larger populations should further explore the comparative health performance of HF than HF × J cows.

**Table 4:** Means ± SE and P-values for annual milk basic composition, health and fertility of 73 individual cows from two breeding groups (100% Holstein-Friesian, HF; 50% Holstein-Friesian:50% Jersey, HF × J) in four low-input dairy farms in England and Wales.

	Breed			
	HF	HF × J	SE	*P*-Value^2^
	n^1^ = 32	n^1^ = 41		
**Health cases per year**				
Mastitis cases	0.53	0.37	0.133	0.228
Lameness cases	0.44	0.39	0.136	0.806
Other Health cases	0.22	0.10	0.059	0.172
All Health cases	1.19	0.85	0.192	0.085
**Fertility**				
Fertility cases (per year)	0.16	0.15	0.046	0.292
Services (number/year to conception)	1.89	1.55	0.138	0.051
Calving interval (days)	383.9	335.6	11.21	0.004
Calving to service interval (days)	88.1	78.6	4.92	0.184

^1^ n is the number of records used to calculate means.

^2^ Significances were declared at *P* < 0.05.

#### Multivariate analyses of the effect of Jersey genetics and diet composition on health and fertility

The RDA biplot showing the relative impact of feed and breed drivers on health and fertility parameters is demonstrated in Fig. 3. Drivers explained 14.9% of the total variation, of which 14.0% was explained by axis 1 and a further 0.9% was explained by axis 2. Jersey accounted for 10.9% and all other drivers explained < 1% of variation each. Fertility treatments were positively associated with intakes of hay and straw and to a lesser extent, grazing and Jersey breed; and negatively associated with intakes of concentrate, minerals and silages. Calving interval, number of services, mastitis cases and other health cases were negatively associated with Jersey genetics. Lameness, all health and calving to first service interval, were positively correlated with concentrate, mineral and silage intakes and negatively associated with hay straw; at a lesser extent, the same response variables were positively correlated with mineral intakes and negatively correlated with grazing intakes.

## Discussion

### Effect of breed on milk production, basic composition and production efficiency

This study, for the first time, assesses the effects of using HF or HF × J in low-input pasture-based systems on FA profiles simultaneously with milk yield and basic composition; efficiency, health and fertility parameters; also accounting for the effect and interactions with season.

In addition, an extensive questionnaire-based data for breeding and feeding practices, accompanying milk samples, allowed for the investigation of the associations between genotype and dietary drivers with productivity, efficiency, health, fertility and milk FA profiles via RDA.

The current study found that HF × J cows produce milk with higher fat and protein concentration; thus agreeing with previous comparisons between HF × J and HF cows^13,20^, which found that milk protein and fat concentrations were higher for HF × J cows (3.12 and 3.83 g/100 milk respectively) compared to HF cows (3.08 and 3.59 g/100 g milk, respectively)^20^. The RDA in the current study suggested that dietary feeds, particularly total forage and grazing intakes, were more important drivers for milk protein content than genetics (although milk protein was the least affected milk component), unlike previous findings showing genetics had a stronger influence on milk solids content than diet^4^.

The current study showed HF × J cows were more efficient converters of feed to milk, fat and protein than HF cows. This agrees with previous studies which found that Jersey cows and HF × J cows produced more fat and protein per kg of total DMI (+ 8 g and + 9 g, respectively comparable with results here) compared to the HF cows^11^; although other studies (in indoor systems relying on TMR) showed no difference^19^. Studies comparing pasture-based Jersey, HF × J and HF also report significantly higher production efficiency for Jersey and HF × J, than HF cows; total DMI per 100 kg BW was 3.99, 3.63 and 3.39 kg, respectively; solids corrected milk per 100 kg of BW was 4.30, 3.95 and 3.41 kg, respectively; milk solids per 100 kg BW was 0.35, 0.32 and 0.27, respectively); and milk solids per 100 kg per kg DMI was 88 g, 87 g and 79 g, respectively)^11^. Similarly, other work investigated production efficiency of HF, HF × J and 3-way crossbred Norwegian Red × HF × J and found that HF × J and Norwegian Red × HF × J produced 10.1% and 3.36% more milk solids per kg metabolic bodyweight (BW^0.75^), respectively, than HF^12^. Studies which observed higher efficiency for HF × J cows have attributed this to higher DMI in relation to BW as a result of a proportionally larger gastrointestinal tract capacity associated with Jersey genetics^10–12^. The smaller size of the crossbred cows could also be associated with an “energy saving” capability, also suggested by Vance et al. (2012), which reduced energy requirement for maintenance and enabled them to produce similar fat and protein yields, while eating less than HF^37^. This allows a greater proportion of energy to be partitioned towards production^37^ and may explain the superior fat and protein efficiency for the HF × J cows in the current study. Additionally, higher feed intake per kg BW may be a result of better grazing behaviour of HF × J compared to HF cows. Other studies found that, in relation to BW, Jersey cows had a higher bite rate, intake rate, grazing time as well as a greater number of mastication’s and faster rate of mastication, when compared to HF^15^. As a result, Jersey cows have been associated with a higher grazing drive^37^. Additionally, when milk fat and protein yield (kg/day) were compared (relative to net energy intake), HF × J required 11% less energy to produce 1 kg of fat and protein, when compared to the HF cows^11^. These results indicate that HF × J cows may be used to increase milk protein and casein contents, as well as improve the conversion of feed (especially grazing) to milk, fat and protein in low-input, pasture-based production systems.

However, the importance of diet in low-input systems should not be overlooked; the RDA in the present study highlighted that diet ingredients were stronger drivers for efficiency parameters than breed, with moist by-products, compound feed and cereals being associated with improved FE and protein efficiency; while, in addition to these drivers, grazing intake was also positively associated with improved fat efficiency. Differences in efficiency can also relate to other aspects of management. Previous work has shown that Jersey cows, in organic systems, are less efficient than in conventional dairy systems by having 10.4% higher feed intakes, without a comparable increase in milk yield. However, these results were from a small meta-analysis dataset^38^. Although previous studies suggested Jersey genetics in organic and conventional low-input systems herds are likely to have lower milk output than HF cows^4^, this was not the case in the present study, where yield was not affected by crossbreeding.

However, it should be noted that dry matter intake in the present study, which is used in the calculation of feed efficiency, has been predicted based on average breed bodyweight and milk yield, as previously shown^3^, because measuring DMI in commercial farms is not feasible. The supply of conserved forage and concentrate feeds (35–40% DMI) at herd level in the present study has been recorded via questionnaires. Predicting DMI (and eventually pasture intake by difference) may have reduced the accuracy of efficiency parameters. In addition, given that previous work shows HF × J crossbreds eat more per kg BW than HF cows (36.3 g vs 33.9 g^11^) this approach may slightly underestimate DMI, although discrepancies, based on the prediction equation used here, would be relatively small, i.e. < 6% or < 1 kg DMI per cow per day. However, results around feed efficiency are in line with previous work in pasture-based animals, which appears to confirm that feed efficiency might be improved by using crossbred cows at pasture-based herds^11^. Although, this may not necessarily be the case for indoor dairy production^19^.

### Effect of breed on milk fatty acid profiles and implications to consumers’ nutrition

The present study found milk from HF × J cows, was higher in SFA than milk from HF cows; thus being in line with previous studies comparing Jersey (or Jersey & Guernsey) milk with other breeds (and in particular Holstein)^39^. A number of studies report HF cows produced milk with less SFA compared with Jersey cows^28,40–43^. Previous studies also found that Jersey milk tends to have a higher proportion of short and medium chained FA^43^. Although, results are variable between studies, some Jersey milk has higher proportions of C4:0, C8:0, C10:0, C12:0, C14:0 and C16:0^42^, whereas others only report higher proportions for C4:0, C6:0 and C8:0^43^. The current study found higher concentration of C4:0, C6:0, C8:0, C10:0, C12:0 and C14:0 for the HF × J compared to the HF milk; in contrast with one study which found that, of all individual SFA quantified, only C16:0 was significantly higher in the HF × J and Jersey milk, when compared to HF milk^28^. Most SFA, with chain lengths up to C14:0, are synthesised de novo in the mammary gland, thus the higher proportion of these short chain FA in milk may suggest HF × J cows have a greater ability for de novo synthesis^43^. This is further supported by the RDA analysis, in the current study, demonstrating a positive association between Jersey genetics and concentrations of C6:0, C8:0, C10:0, C12:0 and C14:0. Although, HF cows had higher desaturase activity markers compared to HF × J. This may also explain the lower C12:0 and C14:0 in the HF breed, since the enzyme desaturase, converts SFAs with between 10 and 18 carbon atoms into MUFA, in the mammary gland^44^ (reducing concentrations of these substrates). This theory is further supported by the higher concentrations of c9 C14:1 and c9 C16:1 in milk from HF cows. The finding that HF × J cows had higher milk C12:0 and C14:0 than HF cows, but no difference for C16:0, concurs with previous reports, that all individual SFA were higher in Jersey and Guernsey milk compared with milk from predominantly Holstein cows, with the exception of C16:0^29,39^.

The higher concentration of SFA in the current study for HF × J milk was due to the higher concentrations of shorter chain SFA between C4:0 and C14:0 and not driven by differences in C16:0 (the major SFA in milk, and the main responsible FA for the assumed increased risk of atherosclerosis, following overconsumption^45^). Although HF × J cows had higher proportions of some nutritionally undesirable individual SFA, they also had a higher proportion of short chain SFA, consistent with previous studies^29,46^, which are considered nutritionally beneficial for human health^47–49^. Consumption of medium chained SFAs (C12:0–C16:0), are considered to increase blood LDL cholesterol linked with atherosclerotic disease^45,50,51^. However, smaller chained SFA (C4:0–C10:0) (present in higher concentrations in HF × J milk, in the current study) have been associated with some human health benefits. Nevertheless, the higher contribution of SFA in HF × J milk, towards dietary reference values (DRV) for SFA, and the higher concentrations of C12:0 and C14:0, which contribute to higher AI and TI and lower HH ratio (considered to increase the atherogenic, thrombogenic and cholesterol-related risks of foods, respectively^44,52–54^, may be considered undesirable from a human health perspective. Previous studies report within pasture-based dairy systems, farms with greater reliance on grazing (organic, low-input), produce milk with a lower AI and TI; and pasture intake is a stronger driver for AI and TI than breed^4^.

The latest UK’s National Diet and Nutrition survey^55^, reports dairy fat intakes of: 13.4 g/d for children 1.5–3.0 years of age, 9.8 g/d for children 4–10 years of age, 8.3 g/d for adolescents 11–18 years of age, 8.8 g/d for adults 19–64 years of age, 9.8 g/d for adults 65–74 years of age and 10.6 g/d for adults 75 + years of age. Based on current nutritional recommendations and dietary reference values (DRV) for SFA intakes^25^, consuming HF × J milk instead of HF milk would increase SFA intake from dairy fats (relative to DRV) from 76.6% to 79.7% for children 1.5–3.0 years of age, from 34.2% to 35.6% for children 4–10 years of age, from 18.9% to 19.6% for adolescents 11–18 years of age, from 20.8% to 21.6% for adults 19–64 years of age, from 26.1% to 27.1% for adults 65–74 years of age and from 29.2% to 30.4% for adults 75 + years of age. Given these relatively small changes, from HF to HF × J dairy fat consumption, along with the fact that ‘SFA’ covers multiple individual FA, some associated with health benefits, it is difficult to conclude if differences in SFA content, between HF or HF × J milk, would impact on human health.

The main *cis* MUFA and *trans*-MUFA in milk are OA and VA, respectively, corresponding here to 69% and 8.7% of total MUFA, respectively. Both OA and VA are associated with beneficial effects on human health. OA lowers overall cholesterol, LDL cholesterol and triacylglycerol^51,56^ and VA is converted to CLA in the cows’ mammary gland, which has been associated with improved cardiovascular health^26^. Previous studies investigating milk OA, report lower concentrations in Jersey (or Guernsey) cows compared to HF cows^29,39^. There have also been differences in proportion of VA reported between breeds. Previous RDA studies, report that milk VA and OA content, is positively associated with grazing and the use of breeds other than HF^4^. However, this differs from other findings, reporting that HF milk had higher concentrations of total C18:1 than Jersey or Brown Swiss cows^43^. The finding of the present study, that HF cows had higher concentrations of OA compared to HF × J cows, is in line with previous works comparing HF to other breeds (Jersey and Guernsey)^42,51,56,57^. Evidence suggests that milk OA may originate directly from cows’ diets ^4^, or is the product of desaturation of C18:0 in the mammary gland via desaturase. In the present study, all desaturase activity indicators (Δ^9^Ι, C14:1/C14:0, C16:1/C16:0, OA/C18:0, RA/VA) were higher in HF cows, compared to HF × J, and a higher Δ^9^-desaturase activity would result in increased OA synthesis and secretion to milk in HF cows. RDA also found a negative association between milk OA and Jersey genetics, while a strong positive association was shown between OA and intakes of grazing, as in previous studies^4,29,43^, total forage and moist by-products. However, the differences between breeding groups in the intakes of these diet ingredients were marginal (lower than 5% DMI) and unlikely to have substantially contributed to the differences in OA.

Total MUFA, represented approximately 69% from OA in the present study, were higher for HF milk than HF × J cows, which agrees with previous findings that milk from Jersey or Guernsey cows contains less MUFAs than other non-specified breeds^39^. Similarly, others reported HF cows produced more total milk MUFA than Jersey cows and Swedish Red × Jersey × HF cows respectively^6,41^. Conversely, other authors report the opposite; Jersey cows produced 0.95 g/dL more MUFA than HF cows^23^. In the current study, milk from HF × J cows contained less c9 C14:1 and c9 C16:1. This could be explained by the higher desaturase activity index for HF cows, as discussed previously^6,28,29^. It has been recommended that enhanced desaturase activity could improve milk quality by increasing MUFA (and PUFA) concentrations in milk^24^ and breeding programs could consider a criterion for desaturase activity (via milk indicators) when selecting for breeding^44^. Potential biomarkers for such purposes have been recommended to be the ratio of product:precursor, or products as a percentage of precursors plus products (*cis*-9 14:1 / 14:0 + cis-9 14:1)^58^. RDA showed there is also an association between MUFA (total, cis, trans) and grazing intake and a strong negative association with grass silage, maize silage and cereals; but differences between breeding groups in the intakes of these diet ingredients in the present study were marginal and unlikely to have substantially affected MUFA.

Based on recent records of UK population dairy fat intakes^55^, nutritional recommendations and DRV for MUFA intakes^25,59^, consuming HF × J milk instead of HF milk would reduce MUFA intakes (relative to DRV) from 26.2% to 24.0% for children 1.5–3.0 years of age, from 12.0% to 11.0% for children 4–10 years of age, from 6.4% to 5.2% for adolescents 11–18 years of age, from 7.1% to 6.5% for adults 19–64 years of age, from 8.9% to 8.2% for adults 65–74 years of age and from 9.8% to 9.0% for adults 75 + years of age. Previous studies reported that replacing SFA with MUFA or *cis* MUFA in human diets lowered CVD markers including serum total cholesterol, LDL cholesterol and total:HDL cholesterol^60,61^. However, given the small differences in the MUFA concentrations between HF and HF × J milk, they do not refer to the main MUFAs associated with human health it is unlikely that these result in any effect on human health.

The main PUFA in milk are LA (the predominant n-6), ALNA (the predominant n-3) and RA (the predominant conjugated FA) which in this study were 21.3%, 15.2% and 23.2% of total PUFA, respectively. LA and ALNA cannot be synthesized in cows’ bodies, but small amounts of dietary LA and ALNA (approximately 20% and 8% of total their intake respectively^62^) escape rumen biohydrogenation, are absorbed in the gut, transferred to the mammary gland and into milk^63^. Milk concentrations of these individual FAs did not differ between HF and HF x J cows in the current study—in agreement with previous work^39^. Studies comparing breeds for milk LA and ALNA concentration demonstrate contrasting outcomes; some found no difference between breeds for ALNA, but LA was higher in HF cows^28^, whereas others found no difference in LA, but HF cows had higher ALNA^42^. Variation could be due to the stronger influence of diet rather than breed. Furthermore, genetic influences on the extent of ruminal biohydrogenation cannot be excluded^63^.

Overall PUFA concentrations were higher in HF milk thus aligning with some previous work^28^. However, one study^39^ found no significant difference between milk from Jersey or Guernsey cows for PUFA compared with non-specified milk, along with other work^41^, reporting no difference in PUFA between Jersey cows and HF cows. RDA analysis identified diet characteristics to be the main influence for milk PUFA concentrations, with grazing intake being the major positive driver; a finding previously reported by others^4,24^. This can be further supported by the fact that cows in the present study produced milk with overall higher concentration of PUFA during the grazing season, including the concentrations of ALNA and RA. The positive association between grazing intake and milk ALNA and RA content may be explained by the fact that 50–75% of fresh grass FA is ALNA, providing a direct source for ALNA but also for the formation of VA in the rumen and its subsequent conversion to RA in the mammary gland^24^.

Ratio of n−3:n−6 was higher in HF × J cows than HF cows in the present study. Although n−3:n−6 ratio is associated with higher grazing intake, and botanically diverse swards^4^, higher n−3:n−6 has also been reported for Jersey and Guernsey milk, compared with non-specified milk and studies suggested that Jersey cows may be more efficient at transferring dietary n-3 from feed to milk than HF cows^39,40^. The present study found that some parameters indicating products of lipid hydrogenation, such as total *trans* FA (excluding FA), were higher in HF milk, which may also indicate a higher rate of n-3 hydrogenation in the rumen of HF cows^40,64^. However, the higher n-3:n-6 in HF × J milk is unlikely to have an impact on human nutrition as the numerical difference when compared with HF milk was marginal (1.06 vs 0.99).

The current study also found that HF cows had higher concentrations of total *trans* FA (excluding VA) and *trans* PUFA concentrations, compared to HF × J cows. Although *trans* FAs are considered to be detrimental, some studies have disputed that *trans* fat from animals, does not increase risk of cardiovascular disease^51^. Despite this, it has been recommended that *trans* FA intake should not exceed 2% food energy^59^. Based on recent records of UK population dairy fat intakes^55^, nutritional recommendations and DRV for *trans* FA intakes^25,59^, and assuming that VA is not included in the *trans* FA sum, consuming HF × J milk instead of HF milk would reduce *trans* FA (excluding VA) intakes (relative to DRV); but an impact to human health should not be expected due to the relatively low changes to the contribution towards DRV (e.g. from 3.5–4.8% to 3.1–4.4% in adults).

Previous work recommends adopting low-input pasture-based dairying as a strategy to increase the beneficial FA (PUFA, n-3, VA, RA, ALNA, EPA, and DPA); and the present study suggests the success of this approach is unlikely be affected by crossbreeding HF with Jersey semen as HF and HF × J cows produced milk with similar concentrations of these FA. Replacing dietary SFA with *cis* PUFA has been well documented in literature, demonstrating beneficial effects on human blood cholesterol^44,61^; but in spite of the differences in total PUFA between HF and HF × J milk, it is interesting to note, their concentrations in *cis*-PUFA did not differ.

### Effect of breed on animal health and fertility

Previous research comparing health and fertility between dairy breeds has shown poorer health and fertility in purebred HF when compared to alternative breeds, such as Jerseys, HF × J^13,14,17,20,21^, New Zealand Friesian cross, Ayrshire Cross, HF × Swedish Red and Shorthorn^5^, Holstein × Simmental^65^, Fleckvieh and Brown Swiss^66^. One study observed more cases of mastitis in HF than HF × J cows^5^ along with other work which reported a 45% higher incidence in HF cows compared to HF × J^17^. It has been surmised that better health and fertility in the HF × J cows may be attributed to their ability to divert energy to restoration of body reserves over body maintenance and growth^19,67^; and recommended that the reduced milk output associated with alternative breeds, may be offset by the possibility of better health and fertility^67^.

However, like other work^12,15^, we found no difference between breeds for mastitis cases. Milk production showed a slightly unfavourable genetic correlation with mastitis and SCC in other studies^68^, but the present work refers to relatively lower yielding animals in pasture-based systems; representing breeding groups with similar milk. Additionally, health challenges seen for housed cows may not necessarily be expected under low-input pasture-based management, since evidence suggests mastitis risk is associated with high intensity conventional systems rather than organic or pasture-based production^69^. RDA in the current study also indicates Jersey genetics were negatively associated with many health and fertility parameters (calving interval, number of services, mastitis cases, other health cases, all health cases and calving to first service interval). This is concurrent with another RDA reporting a negative association between mastitis and other veterinary treatments and the introduction of alternative cow breed genetics and pasture intake^4^.

A previous study found HF × J cows had higher pregnancy rates to first service, higher in-calf rates after 6 and 13 weeks breeding than pure HF cows^10^. We found HF × J cows had a shorter calving interval than the HF cows, in agreement with other work^14^. In any block calving herd, good fertility and a short calving interval is crucial for profitability, thus HF × J cows in such seasonal production systems may benefit financial sustainability. RDA results here also suggested Jersey genetics were negatively associated with poor fertility parameters such as number of services, calving to first service interval. Evidence also suggests that excessive negative energy balance and weight loss in early lactation is associated with poor fertility^70^ and HF × J cows have a superior ability to partition energy toward restoring body reserves compared to HF cows^46^. Previous work has presumed that the fitter body condition of HF × J cows could be responsible for improved health and fertility characteristics^20^.

## Conclusions

In the current study, crossbred Holstein–Friesian (HF) × Jersey (J) cows had superior fat, protein and casein content in milk, with total yields being similar to that of HF cows; parameters considered beneficial for economic performance, on low-input dairy farms, particularly when milk payments rely on fat and protein content, in addition to milk volume. Feed, fat and protein efficiency was higher in HF × J cows, but care should be taken when interpreting this result, as they rely on predicted feed intake; although this finding is in line with previous work. However, the HF × J cows produced milk with more SFA content compared to HF cows although, not as a result of differences in nutritionally undesirable C16:0 but due to short chain SFA (C4:0–C10:0) considered beneficial to human health. However, overall differences in the milk fatty acid profile between HF and HF × J cows were relatively small especially considered against likely consumer intakes and are unlikely to affect human health. HF × J cows had better fertility with a shorter calving interval compared to HF cows, also relevant for profitability in dairy production, particularly in low-input block-calving production systems. Whilst there were no other differences between breeds for health or fertility parameters, the redundancy analysis showed a negative association between Jersey genetics and grazing with recorded health incidences. This study demonstrated that low-input dairy farming strategies can benefit in some ways (efficiency, fertility) by using alternative breeds in crossbreeding schemes.

## Supplementary Information

Supplementary Information.

## Data Availability

The datasets used and/or analysed during the current study are available from the corresponding author on reasonable request.

## Abbreviations

ALNA: α-Linolenic acid
AI: Atherogenicity index
BW: Body weight
C4:0: Butyric acid
C10:0: Capric acid
C6:0: Caproic acid
C8:0: Caprylic acid
CVD: Cardiovascular disease
cMUFA: *cis*-monounsaturated fatty acid
cPUFA: *cis*-polyunsaturated fatty acid
CLA: Conjugated linoleic acid
DRV: Dietary reference values
DPA: Docosapentaenoic acid
DMI: Dry matter intake
EPA: Eicosapentaenoic acid
FA: Fatty acid
FE: Feed efficiency
HDL: High density lipoprotein
HF: Holstein-Friesian
HF × J: Holstein-Friesian × Jersey
C12:0: Lauric acid
LDL: Low density lipoprotein
MUFA: Monounsaturated fatty acid
RDA: Multivariate redundancy analysis
C14:0: Myristic acid
OA:0: Oleic acid
n-3: Omega-3 polyunsaturated fatty acid
n-6: Omega-6 polyunsaturated fatty acid
C16:0: Palmitic acid
PUFA: Polyunsaturated fatty acid
REML: Residual maximum likelihood analysis
RA: Rumenic acid
SFA: Saturated fatty acid
SCC: Somatic cell count
C18:0: Stearic acid
TI: Thrombogenicity index
VA: Vaccenic acid
