# Supplementary material for: Performance and milk quality parameters of Jersey crossbreds in low-input dairy systems
Source: Sci Rep. 2022 May 9;12:7550. doi: 10.1038/s41598-022-10834-4 (PMC9085769; doi:10.1038/s41598-022-10834-4)
Supplement: Supplementary file 1 — Supplementary Information. [file 41598_2022_10834_MOESM1_ESM.pdf]

SUPPLEMENTARY INFORMATION

Table S1. Means for the estimated dietary components (% of DMI) of 73 individual cows from 4 low-input farms across 3 seasons in England

	**Farm 1**		**Farm 2**		**Farm 3**		**Farm 4**	
	**n[1]=25**		**n[1]=19**		**n[1]=24**		**n[1]=5**	
	HF	HF×J	HF	HF×J	HF	HF×J	HF	HF×J
Number of animals per breed	n=12	n=13	n=10	n=9	n=8	n=16	n=2	n=3
***Diet components (% DMI unless otherwise stated)*[2]**								
	**n[3]=57**		**n[3]=52**		**n[3]=52**		**n[3]=15**	
Grazing	70.8		60.1		46.9		86.2	
Total forage	81.5		81.6		82.4		93.6	
Grass silage	0.0		12.9		21.5		6.4	
Maize silage	10.7		8.6		12.0		0.0	
Wholecrop	0.0		0.0		2.0		0.0	
Hay/Straw	0.0		0.0		0.0		0.9	
Moist by products	6.4		1.2		1.8		0.0	
Dry straight feeds	12.1		0.0		3.7		0.0	
Cereals	0.0		0.0		0.0		6.4	
Compound	0.0		17.3		12.1		0.0	
Minerals/Vitamins (g/cow/day)	120		0.0		40		0.0	

[1] The number of individual animals in each farm.
[2]The average intake expressed as % DMI across the experiment.
[3]The number of records used to calculate means.

Table S2. Means ± SE and P-values for breed and season on the fatty acid profile of milk collected from 73 individual cows from two breeding groups (100% Holstein-Friesian, HF; 50% Holstein-Friesian:50% Jersey, HF×J) and different seasons in four low-input dairy farms in England and Wales

	Breed				Season					Breed × Season
	HF	HF×J			Spring	Summer	Autumn			
	n[1]=83	n[1]=95	SE	P-value[2]	n[1]=58	n[1]=66	n[1]=54	SE	P-Value[2]	P-value[2]
Individual FA (g/kg total FA)										
C4:0	27.2	29.8	0.66	0.015	24.3[b]	31.3[a]	29.9[a]	0.73	<0.001	0.285
C5:0	0.28	0.27	0.014	0.810	0.35[a]	0.22[b]	0.25[b]	0.015	<0.001	0.672
C6:0	25.7	27.6	0.44	0.001	27.9[a]	25.6[b]	27.0[a]	0.53	<0.001	0.217
C7:0	0.32	0.29	0.022	0.911	0.46[a]	0.20[b]	0.26[b]	0.022	<0.001	0.625
C8:0	13.6	14.9	0.29	<0.001	16.0[a]	13.5[b]	13.4[b]	0.33	<0.001	0.398
C9:0	0.37	0.35	0.028	0.881	0.57[a]	0.25[b]	0.29[b]	0.027	<0.001	0.605
C10:0	30.37	33.39	0.829	0.002	36.9[a]	29.3[b]	30.0[b]	0.93	<0.001	0.546
C11:0	2.93	2.89	0.082	0.965	3.01[a]	2.54[b]	3.25[a]	0.093	<0.001	0.291
C12:0	36.4	40.1	1.03	<0.001	45.7[a]	31.6[c]	38.7[b]	1.02	<0.001	0.515
C13:0	2.01	1.94	0.085	0.921	2.43[a]	1.48[c]	2.09[b]	0.087	<0.001	0.491
C14:0	115.0	119.6	1.57	0.011	117.1[b]	112.4[c]	124.0[a]	1.83	<0.001	0.821
c9 C14:1	10.7	9.49	0.356	<0.001	8.91[b]	9.05[b]	12.5[a]	0.38	<0.001	0.424
C15:0	12.4	11.5	0.23	0.015	12.0	12.0	11.7	0.28	0.773	0.601
c9 C15:1	0.14	0.11	0.008	0.002	0.13[b]	0.08[c]	0.15[a]	0.009	<0.001	0.166
C16:0	306.8	311.8	5.71	0.356	276.0[c]	292.1[b]	366.7[a]	4.71	<0.001	0.75
t9 C16:1	4.82	4.52	0.145	0.041	4.52[b]	5.58[a]	3.69[c]	0.148	<0.001	0.434
c9 C16:1	20.1	17.3	0.54	<0.001	17.2[b]	17.4[b]	21.6[a]	0.61	<0.001	0.277
C17:0	5.08	4.93	0.082	0.016	4.52[c]	5.62[a]	4.76[b]	0.010	<0.001	0.429
c9 C17:1	2.20	1.84	0.058	<0.001	2.15[a]	1.90[b]	1.98[ab]	0.073	0.030	0.985
C18:0	94.0	100.2	2.55	0.186	94.1[b]	112.2[a]	82.6[c]	2.68	<0.001	0.375
t6+t7+t8 C18:1	1.93	1.82	0.071	0.342	2.45[a]	1.56[b]	1.63[b]	0.066	<0.001	0.278
t9 C18:1	1.51	1.36	0.056	0.074	1.89[a]	1.13[c]	1.30[b]	0.052	<0.001	0.197
t10 C18:1	3.31	2.67	0.287	0.138	5.36[a]	1.63[b]	2.03[b]	0.211	<0.001	0.391
t11 C18:1 (VA)	22.9	23.3	1.51	0.962	27.3[a]	29.4[a]	11.0[b]	1.50	<0.001	0.313

t12+t13+t14 C18:1	2.95	2.67	0.117	0.104	3.76[a]	2.06[c]	2.68[b]	0.103	<0.001	0.602
c9 C18:1 (OA)	191.9	174.6	4.18	0.001	187.9[a]	197.1[a]	159.3[b]	4.78	<0.001	0.887
t15 C18:1	2.59	2.34	0.130	0.222	3.48[a]	2.06[b]	1.84[b]	0.128	<0.001	0.954
c11 C18:1	3.61	3.47	0.101	0.302	3.94[a]	3.41[b]	3.24[b]	0.118	<0.001	0.336
c12 C18:1	0.95	0.89	0.061	0.472	1.29[a]	0.56[c]	0.95[b]	0.058	<0.001	0.144
c13 C18:1	0.57	0.50	0.034	0.070	0.81[a]	0.44[b]	0.35[b]	0.032	<0.001	0.515
c14+t16 C18:1	3.51	3.37	0.155	0.834	4.72[a]	3.08[b]	2.49[c]	0.145	<0.001	0.672
c15 C18:1	1.39	1.25	0.058	0.127	1.74[a]	1.26[b]	0.93[c]	0.059	<0.001	0.736
t11t15 C18:2	0.33	0.32	0.016	0.992	0.30	0.33	0.34	0.019	0.293	0.128
t10t14 C18:2	0.43	0.43	0.033	0.784	0.56[a]	0.52[a]	0.19[b]	0.035	<0.001	0.717
c9t13 C18:2	0.92	0.77	0.077	0.427	1.39[a]	0.40[c]	0.79[b]	0.064	<0.001	0.14
t9t12 C18:2	0.97	0.77	0.044	0.002	1.19[a]	0.65[c]	0.78[b]	0.046	<0.001	0.131
t8c13 C18:2	2.12	1.42	0.216	0.025	3.27[a]	1.14[b]	0.85[b]	0.183	<0.001	0.013
c9t12 C18:2	1.28	1.03	0.052	0.001	1.51[a]	1.01[b]	0.92[b]	0.057	<0.001	0.248
t9c12 C18:2	0.46	0.43	0.027	0.945	0.70[a]	0.35[b]	0.30[b]	0.023	<0.001	0.692
ct1014+1216 C18:2	0.46	0.44	0.027	0.402	0.61[a]	0.50[b]	0.21[c]	0.024	<0.001	0.322
t11c15 C18:2	3.13	3.05	0.196	0.774	3.44[b]	4.20[a]	1.35[c]	0.178	<0.001	0.79
c9c12 C18:2 (LA)	10.1	9.55	0.421	0.233	13.2[a]	7.15[c]	9.38[b]	0.381	<0.001	0.301
c10 C19:1	1.03	0.90	0.032	0.003	1.10[a]	0.94[b]	0.84[b]	0.037	<0.001	0.306
Unidentified C18:2 (LA3)	0.89	0.79	0.019	<0.001	0.73[b]	0.90[a]	0.88[a]	0.022	<0.001	0.122
unknown C18:2 (LA2)	0.54	0.54	0.013	0.271	0.59[a]	0.58[a]	0.46[b]	0.014	<0.001	0.918
c9c15 C18:2	0.20	0.18	0.011	0.115	0.27[a]	0.15[b]	0.16[b]	0.011	<0.001	0.491
c12c15 C18:2	0.11	0.10	0.008	0.421	0.14[a]	0.10[b]	0.07[c]	0.009	<0.001	0.643
C20:0	1.08	1.12	0.027	0.297	0.97[b]	1.20[a]	1.13[a]	0.031	<0.001	0.527
c6c9c12 C18:3 (GLA)	0.16	0.14	0.007	0.070	0.20[a]	0.12[b]	0.13[b]	0.008	<0.001	0.031
c5 C20:1	0.00	0.02	0.006	0.133	0.00	0.00	0.03	0.006	0.091	0.107
c8 C20:1	0.83	0.77	0.034	0.469	0.62[b]	0.84[a]	0.94[a]	0.036	<0.001	0.765
c9c12c15 C18:3 (ALN)	6.95	7.01	0.256	0.992	8.51[a]	7.63[b]	4.56[c]	0.224	<0.001	0.608
c9t11 18:2 (CLA9)	11.3	10.1	0.70	0.150	11.9[a]	13.2[a]	6.26[b]	0.762	<0.001	0.576
t11c13 CLA	0.35	0.36	0.027	0.865	0.38[b]	0.48[a]	0.18[c]	0.028	<0.001	0.895
unknown CLA1	0.27	0.29	0.012	0.576	0.22[b]	0.37[a]	0.23[b]	0.012	<0.001	0.894
unknown tt CLA2	0.41	0.33	0.031	0.047	0.60[a]	0.31[b]	0.18[c]	0.027	<0.001	0.07

unknown tt CLA3	0.60	0.57	0.044	0.910	0.86[a]	0.63[b]	0.22[c]	0.039	<0.001	0.259
unknown tt CLA6	0.24	0.22	0.014	0.481	0.24[a]	0.27[a]	0.17[c]	0.016	<0.001	0.571
c9c13c15 C18:3	0.32	0.33	0.023	0.990	0.32[a]	0.46[a]	0.17[b]	0.023	<0.001	0.713
c11c14 C20:2	0.26	0.25	0.007	0.457	0.21[c]	0.28[a]	0.26[b]	0.008	<0.001	0.813
c9c11c15 C18:3	0.58	0.56	0.034	0.840	0.70[a]	0.68[a]	0.31[b]	0.034	<0.001	0.408
C22:0	0.57	0.60	0.020	0.484	0.46[c]	0.75[a]	0.51[b]	0.018	<0.001	0.911
c8c11c14 C20:3	0.58	0.50	0.021	0.006	0.65[a]	0.46[c]	0.52[b]	0.023	<0.001	0.032
c13 C22:1	0.13	0.12	0.007	0.063	0.09[c]	0.15[a]	0.13[b]	0.007	<0.001	0.426
c11c14c17 C20:3	0.20	0.22	0.010	0.541	0.20	0.22	0.21	0.010	0.260	0.541
c5c8c11c14 C20:4	0.83	0.72	0.027	0.006	0.91[a]	0.70[b]	0.70[b]	0.031	<0.001	0.115
C23:0	0.30	0.33	0.011	0.032	0.26[c]	0.36[a]	0.31[b]	0.012	<0.001	0.715
c13c16 C22:2	0.66	0.69	0.028	0.079	0.74[b]	0.83[a]	0.41[c]	0.025	<0.001	0.422
c5c8c11c14c17 C20:5 (EPA)	0.80	0.79	0.026	0.493	0.89[a]	0.87[a]	0.61[b]	0.026	<0.001	0.493
C24:0	0.61	0.58	0.026	0.805	0.72[a]	0.58[b]	0.48[c]	0.027	<0.001	0.144
c15 C24:1	0.14	0.17	0.008	<0.001	0.18[a]	0.19[a]	0.10[b]	0.008	<0.001	0.576
c13c16c19 C22:3	0.09	0.10	0.005	0.118	0.08[b]	0.10[a]	0.09[b]	0.005	<0.001	0.699
c7c10c13c16 C22:4	0.09	0.11	0.010	0.103	0.09	0.09	0.12	0.012	0.383	0.153
c7c10c13c16c19 C22:5 (DPA)	1.15	1.12	0.035	0.857	1.18[a]	1.18[a]	1.01[b]	0.041	0.003	0.813
c7c10c13c16c19 C22:6 (DHA)	0.08	0.09	0.006	0.249	0.10[a]	0.09[b]	0.07[b]	0.008	0.003	0.233

[1]n is the number of records used to calculate means. Records with missing data were not included in the means ± SE and P-values calculations.

[2]Significances were declared at P<0.05. Means for season within a row with different lower-case letters are significantly different according to Fisher's Least Significant Difference test (P<0.05).

Table S3. Means ± SE and P-values for breed × season interaction for yield, basic composition and efficiency parameters of milk collected from 73 individual cows from two breeding groups (100% Holstein-Friesian, HF; 50% Holstein-Friesian:50% Jersey, HF×J) and different seasons in four low-input dairy farms in England and Wales

	HF			HF × J			SE	P-value[2]
	Spring n=29[1]	Summer n=20	Autumn n=29	Spring n=30	Summer n=34	Autumn n=30		
Productivity (kg/cow/day)								
Milk yield	29.4	19.0	15.1	29.2	19.4	13.2	1.55	0.804
Milk fat yield	1.15	0.68	0.56	1.18	0.70	0.56	0.059	0.900
Milk protein yield	0.98	0.61	0.52	1.05	0.65	0.52	0.047	0.613
Basic Composition (g/kg milk)								
Milk fat	3.97	3.66	3.89	4.34	4.00	4.23	0.254	0.959
Milk protein	3.38	3.26	3.67	3.67	3.52	4.09	0.116	0.841
Milk casein[3]	*	2.38	2.60	*	2.42	3.03	0.114	0.038
Milk whey protein[3]	*	0.79	0.91	*	0.79	0.85	0.058	0.693
Fat:protein (g/g)	1.18	1.12	1.06	1.17	1.12	1.05	0.060	0.916
Milk lactose (g/kg milk)	4.80	4.43	4.25	4.81	4.55	4.31	0.097	0.863
Urea (g/l milk)	0.02	0.03	0.01	0.03	0.02	0.01	0.002	0.235
Milk SCC (x1000/ml milk)	206	117	196	89.1	258	141	54.3	0.386
Efficiency Parameters								
Feed efficiency (kg milk/kg DMI)	1.46	1.01	0.81	1.65	1.17	0.85	0.074	0.474
Feed non-grazing efficiency (kg milk/kg non-grazing DMI)	10.8	12.3	1.23	9.94	9.75	1.10	1.513	0.907
Feed concentrate efficiency (kg milk/kg concentrate DMI)	7.38	14.1	5.23	6.86	12.3	4.38	0.836	0.836
Fat efficiency (g fat/kg DMI)	57.2	35.8	30.4	68.2	43.3	35.7	2.98	0.512
Fat non-grazing efficiency (g fat/kg non-grazing DMI)	462	512	47.4	483	410	47.9	54.83	0.939
Fat concentrate efficiency (g fat/kg concentrate DMI)	281	578	202	278	499	182	32.0	0.831
Protein efficiency (g protein/kg DMI)	48.8	32.3	28.2	60.0	39.6	33.5	2.17	0.206
Protein non-grazing efficiency (g protein/kg non-grazing DMI)	355	463	44.5	369	347	45.2	41.79	0.661
Protein concentrate efficiency (g protein/kg concentrate DMI)	246	524	182	251	446	173	25.9	0.595

[1]n is the number of records used to calculate means ± SE and P-values. Records with missing values were not included in the analysis. Data for milk casein and whey protein were not collected for spring

[2] Significances were declared at P < 0.05. Means within a row with different upper-case letters are significantly different according to Fisher's protected least significant difference test (P < 0.05).

Table S4. Means ± SE and P-values for breed × season interaction for FA profile of milk collected from 73 individual cows from two breeding groups (100% Holstein-Friesian, HF; 50% Holstein-Friesian:50% Jersey, HF×J) and different seasons in four low-input dairy farms in England and Wales

	HF			HF × J			SE	P-value[2]
	Spring n=28[1]	Summer n=28	Autumn n=27	Spring n=30	Summer n=38	Autumn n=27		
Individual FA (g/kg total FA)								
SFA								
C4:0	2.36	3.01	2.79	2.48	3.22	3.20	0.100	0.285
C6:0	2.71	2.47	2.54	2.86	2.62	2.85	0.072	0.217
C8:0	1.53	1.30	1.26	1.67	1.39	1.42	0.045	0.398
C10:0	3.49	2.81	2.80	3.87	3.02	3.21	0.129	0.546
C12:0	4.31	3.02	3.59	4.81	3.27	4.15	0.140	0.515
C14:0	11.5	11.0	12.1	11.9	11.5	12.8	0.255	0.821
C16:0	27.4	28.7	36.0	27.7	29.6	37.3	0.666	0.750
C18:0	8.88	11.0	8.26	9.91	11.4	8.26	0.375	0.375
MUFA								
VA	2.58	3.03	1.22	2.87	2.87	0.99	0.211	0.313
OA	19.8	20.7	17.0	17.9	19.0	14.8	0.654	0.887
PUFA								
LA	1.32	0.70	1.00	1.32	0.73	0.87	0.053	0.301
RA	1.23	1.41	0.75	1.15	1.25	0.50	0.107	0.576
ALNA	0.87	0.76	0.44	0.83	0.76	0.47	0.032	0.608
EPA	0.09	0.09	0.06	0.09	0.08	0.06	0.004	0.493
DPA	0.12	0.12	0.10	0.12	0.12	0.10	0.006	0.813
DHA	0.01	0.01	0.01	0.01	0.01	0.01	0.001	0.233
FA groups (g/kg total FA)								
SFA[3]	64.9	65.9	71.8	67.7	68.4	75.6	0.914	0.674
MUFA[4]	29.1	29.3	24.6	26.8	27.0	21.3	0.793	0.681
cis MUFA[6]	24.1	24.6	21.85	21.7	22.6	19.0	0.680	0.766
trans MUFA[7]	5.06	4.63	2.78	5.16	4.39	2.30	0.250	0.454
PUFA[5]	5.96	4.82	3.54	5.43	4.60	3.07	0.175	0.586
cis PUFA[8]	2.94	2.17	1.98	2.84	2.17	1.86	0.075	0.639
trans PUFA[9]	0.46	0.34	0.26	0.40	0.33	0.22	0.016	0.479
cis/trans + *trans/cis* PUFA[10]	2.56	2.31	1.30	2.19	2.10	0.98	0.132	0.731
n-3[11]	1.66	1.62	0.89	1.57	1.59	0.90	0.058	0.708
n-6[12]	2.00	1.16	1.45	1.89	1.17	1.26	0.062	0.322
n-3/n-6 ratio	0.89	1.43	0.63	0.91	1.41	0.73	0.057	0.727
n-6/n-3 ratio	1.23	0.74	1.72	1.23	0.78	1.56	0.068	0.327
trans FA[13]	5.52	4.98	3.05	5.55	4.71	2.53	0.256	0.504
trans FA (exc. VA)	2.94	1.95	1.83	2.68	1.84	1.54	0.093	0.762
Human health related indices								
AI[14]	2.27	2.31	3.31	2.54	2.58	4.05	0.133	0.135
TI[15]	2.27	2.44	3.63	2.51	2.66	4.22	0.116	0.230
HH[16]	0.61	0.62	0.42	0.53	0.55	0.35	0.023	0.992
Δ^9-desaturase activity indicators								
Δ^9I[16]	0.32	0.32	0.27	0.29	0.29	0.24	0.008	0.786

C14:1/C14:0	0.09	0.09	0.11	0.07	0.08	0.09	0.005	0.450
C16:1/C16:0	0.07	0.07	0.06	0.06	0.06	0.05	0.003	0.567
OA/C18:0	2.30	1.88	2.30	1.85	1.69	1.88	0.086	0.171
RA/VA	0.49	0.46	0.64	0.41	0.43	0.50	0.022	0.032

[1]n is the number of records used to calculate means

[2] Significances were declared at P < 0.05. Means within a row with different upper-case letters are significantly different according to Fisher's protected least significant difference test (P < 0.05).

[3]SFA: C4:0, C5:0, C6:0, C7:0, C8:0, C9:0, C10:0, C11:0, C12:0, C13:0, C14:0, C15:0, C16:0, C17:0, C18:0, C20:0, C22:0, C24:0

[4]MUFA: c9 C14:1, c9 C15:1, t9 C16:1, c9 C16:1, c9 C17:1, t6+t7+t8 C18:1, t9 C18:1, t10 C18:1, t11 C18:1, t12+t13+t14 C18:1, c9 C18:1 (OA), t15 C18:1, c11 C18:1, c12 C18:1, c13 C18:1, c14+t16 C18:1, c15 C18:1, c15 C18:1, c10 C19:1, c5 C20:1, c8 C20:1, c13 C22:1, c15 C24:1

[5]PUFA: t11t15 C18:2, t11t15 C18:2, t10t14 C18:2. C9t13 C18:2, t9t12 C18:2, t8c13 C18:2, c9t12 C18:2, t9c12 C18:2, t11c15 C18:2, c9c12 C18:2 (LA), two unidentified C18:2, c9c15 C18:2, c12c15 C18:2, c6c9c12 C18:3 (GLA), c9c12c15 C18:3 (ALN), c9t11 18:2 (RA), four unidentified conjugated linoleic acid (CLA) isomers, t11c13 C18:2
CLA, c9c13c15 C18:3, c11c14 C20:2, c9c11c15 C18:3, c8c11c14 C20:3, c11c14c17 C20:3, c5c8c11c14 C20:4, c13c16 C22:2, c5c8c14c17 C20:5 (EPA), c13c16c19 C22:3, c7c10c13c16 C22:4, c7c10c13c16c19 C22:5 (DPA), c7c10c13c16c19 C22:6 (DHA)

[6]*cis* MUFA: c9 C14:1, c9 C15:1, c9 C16:1, c9 C17:1, c9 C18:1 (OA), c11 C18:1, c12 C18:1, c13 C18:1, c14 C18:1, c15 C18:1, c10 C19:1, c5 C20:1, c8 C20:1, c13 C22:1, c15 C24:1

[7]*trans* MUFA: t9 C16:1, t6+t7+t8 C18:1, t9 C18:1, t10 C18:1, t11 C18:1 (VA), t12+t13+t14 C18:1, t15 C18:1, t16 C18:1

[8]*cis*PUFA: c9c12 C18:2 (LA), unidentified *cis/cis* C18:2, c9c15 C18:2, c12c15 C18:2, c6c9c12 C18:3 (GLA), c9c12c15 C18:3 (ALN), c9c13c15 C18:3, c11c14 C20:2, c9c11c15 C18:3, c8c11c14 C20:3, c11c14c17 C20:3, c5c8c11c14 C20:4, c13c16 C22:2, c5c8c11c14c17 C20:5 (EPA), c13c16c19 C22:3, c7c10c13c16 C22:4, c7c10c13c16c19 C22:5 (DPA), c7c10c13c16c19 C22:6 (DHA)

[9]*trans* PUFA:t11t15 C18:2, t10t14 C18:2, t9t12 C18:2, unidentified *trans/trans* C18:2, unidentified *trans/trans* CLA isomers

[10]*cis/trans+trans/cis* PUFA: c9t13 C18:2, t8c13 C18:2, c9t12 C18:2, t9c12 C18:2, ct1014+1216 C18:2, t11c15 C18:2, unidentified *cis/trans+trans/cis* C18:2, RA, t11c13 CLA, unidentified *cis/trans+trans/cis* CLA

[11]omega-3 PUFA (n-3): t11t15 C18:2, t11c15 C18:2, c9c15 C18:2, c12c15 C18:2, c9c12c15 C18:3 (ALN), c9c13c15 C18:3, c9c11c15 C18:3, c11c14c17 C20:3, c5c8c11c14c17 C20:5 (EPA), c13c16c19 C22:3, c7c10c13c16c19 C22:5 (DPA), c7c10c13c16c19 C22:6 (DHA)

[12]omega-6 PUFA(n-6): t9t12 C18:2, c9t12 C18:2, t9c12 C18:2, c9c12 C18:2 (LA), c6c9c12 C18:3 (GLA), c11c14 C20:2, c8c11c14 C20:3, c5c8c11c14 C20:4, c13c16 C22:2, c7c10c13c16 C22:4

[13]*trans* FA: t9 C16:1, t6+t7+t8 C18:1, t9 C18:1, t10 C18:1, t11 C18:1 (VA), t12+t13+t14 C18:1, t15 C18:1, c14+t16 C18:1, t9 C16:1, t6+t7+t8 C18:1, t9 C18:1, t10 C18:1, t11 C18:1 (VA), t12+t13+t14 C18:1, t15 C18:1, t11t15 C18:2, t10t14 C18:2, t9t12 C18:2, t11c15 C18:2, t11t15 C18:2, t10t14 C18:2, t9t12 C18:2, unidentified *trans/trans* C18:2, unidentified *trans/trans* CLA

[14]Atherogenicity index=(C12:0+4×C14:0+C16:0)/(MUFA+PUFA), as described in Srednicka-Tober et al. (2016)

[15]Thrombogenicity index=(C14:0+C16:0+C18:0)/[(0.5×MUFA)+(0.5×n-6)+(3×n-3)+(n-3/n-6)] as described in Srednicka-Tober et al. (2016)

[16]Hypocolesterolemic to hypercolesteremic ratio=(C18:1 *cis*9 + total PUFA) / (C12:0 + C14:0 + C16:0)

[17]Δ9-desaturase activity index= (c9 C14:1+c9 C16:1+OA+RA)/(c9C14:1+c9 C16:1+OA+RA+C14:0+C16:0+C18:0+VA) as described in Kay et al. (2004)
